# Progressive colonization and restricted gene flow shape island-dependent population structure in Galápagos marine iguanas (*Amblyrhynchus cristatus*)

**DOI:** 10.1186/1471-2148-9-297

**Published:** 2009-12-22

**Authors:** Sebastian Steinfartz, Scott Glaberman, Deborah Lanterbecq, Michael A Russello, Sabrina Rosa, Torrance C Hanley, Cruz Marquez, Howard L Snell, Heidi M Snell, Gabriele Gentile, Giacomo Dell'Olmo, Alessandro M Powell, Adalgisa Caccone

**Affiliations:** 1 Department of Ecology and Evolutionary Biology and Yale Institute for Biospheric Studies - Molecular Systematics and Conservation Genetics Laboratory, New Haven, Connecticut 06511, USA; 2 Current address: Department of Animal Behaviour, University of Bielefeld, D-33501 Bielefeld, Germany; 3 Current address: Marine Biology Laboratory, University of Mons-Hainaut, 7000 Mons, Belgium; 4 Department of Biology, University of British Columbia Okanagan, Kelowna, British Columbia V1V 1V7, Canada; 5 Galápagos National Park, Puerto Ayora, Galápagos, Ecuador; 6 Museum of Southwestern Biology, Department of Biology, University of New Mexico, Albuquerque, New Mexico 87131, USA; 7 Department of Biology, Tor Vergata University, 00133 Rome, Italy; 8 Ornis italica, Piazza Crati 15, 00199 Rome, Italy; 9 36 Carew Rd, Hamden, CT 06517, USA

## Abstract

**Background:**

Marine iguanas (*Amblyrhynchus cristatus*) inhabit the coastlines of large and small islands throughout the Galápagos archipelago, providing a rich system to study the spatial and temporal factors influencing the phylogeographic distribution and population structure of a species. Here, we analyze the microevolution of marine iguanas using the complete mitochondrial control region (CR) as well as 13 microsatellite loci representing more than 1200 individuals from 13 islands.

**Results:**

CR data show that marine iguanas occupy three general clades: one that is widely distributed across the northern archipelago, and likely spread from east to west by way of the South Equatorial current, a second that is found mostly on the older eastern and central islands, and a third that is limited to the younger northern and western islands. Generally, the CR haplotype distribution pattern supports the colonization of the archipelago from the older, eastern islands to the younger, western islands. However, there are also signatures of recurrent, historical gene flow between islands after population establishment. Bayesian cluster analysis of microsatellite genotypes indicates the existence of twenty distinct genetic clusters generally following a one-cluster-per-island pattern. However, two well-differentiated clusters were found on the easternmost island of San Cristóbal, while nine distinct and highly intermixed clusters were found on youngest, westernmost islands of Isabela and Fernandina. High mtDNA and microsatellite genetic diversity were observed for populations on Isabela and Fernandina that may be the result of a recent population expansion and founder events from multiple sources.

**Conclusions:**

While a past genetic study based on pure *F*_ST _analysis suggested that marine iguana populations display high levels of nuclear (but not mitochondrial) gene flow due to male-biased dispersal, the results of our sex-biased dispersal tests and the finding of strong genetic differentiation between islands do not support this view. Therefore, our study is a nice example of how recently developed analytical tools such as Bayesian clustering analysis and DNA sequence-based demographic analyses can overcome potential biases introduced by simply relying on *F*_ST _estimates from markers with different inheritance patterns.

## Background

The legacy of the Galápagos archipelago may be forever attached to the development of Darwin's theory. Yet, the unique character of these islands have continued to make them an ideal model for evolutionary study [1,2]. The Galápagos archipelago is geographically isolated - approximately 1,000 km west of South America - and has never been attached to any continental land mass [3]. Consequently, it is home to many endemic taxa that have colonized the islands either once [4-10] or very few times [9,11,12]. Such a system offers an opportunity to study the radiation of species from a limited ancestral stock without the confounding signals of recurrent colonization. In addition, the islands vary significantly in size and degree of isolation, providing a range of conditions under which to examine the interplay between evolutionary diversification and different demographic processes [13,14]. Finally, the geologic history of the Galápagos is well known, supplying a temporal framework upon which to reconstruct the biogeographic history of various species. The islands were produced by a hotspot that lies beneath the Nazca plate, which is traveling in an eastward direction. Consequently, island ages generally decrease from east to west [3,15-17], and the colonization sequences of many organisms show a progression from older to younger islands (i.e. progression rule or "island progression hypothesis" sensu Wagner and Funk [18]; reviewed in [2]).

Among the organisms that inhabit the Galápagos, one of the most amenable to evolutionary study is the marine iguana (*Amblyrhynchus cristatus*). This species is endemic to the archipelago and is frequently found along the coasts of all the major islands as well as many smaller ones. Marine iguanas exhibit a unique natural history among lizards, feeding almost exclusively on specific algae species in the intertidal or subtidal zones, while breeding and nesting completely on land [19,20]. They possess physical attributes that enable them to negotiate the marine environment, including a flattened tail for swimming and long, sharp nails for clinging to rocks in the surf. The presence of marine iguana populations on islands throughout the archipelago provides a rich system for examining the roles of island population size, age and isolation, as well as current flow, on patterns of migration, distribution, and long-term population history.

Morphological and genetic data have revealed that the closest relative of *Amblyrhynchus *is the genus of terrestrial iguanas, *Conolophus*, which is also endemic to the Galápagos [8,21-23]. Molecular dating based on mitochondrial DNA (mtDNA) and immunological comparisons yielded a divergence time estimate of 10-20 million years (myr) for the two genera [8,24], which is significantly more ancient than the 3-5 myr age estimate of the oldest islands [3,15,16]. A proposed explanation for these results is that marine and land iguanas diverged from each other on now-sunken islands lying to the east of the present-day archipelago [8,24-26].

A comprehensive genetic study based on both mitochondrial cytochrome b (cyt*b*) data as well as nuclear-coded loci (three microsatellite and three minisatellite loci), traced back the microevolution of 22 population/subpopulations of marine iguanas from 15 islands in the archipelago [27]. Despite at least ten million years of independent evolutionary history on the Galápagos, this study suggested that only one or a few related mtDNA haplotypes were involved in the colonization of the present-day archipelago from now-submerged islands [27]. While the overall levels of genetic divergence at both mtDNA and nuclear markers were low, these two markers revealed different patterns of genetic structuring and migration between island populations. Based on cyt*b *data, marine iguana populations were grouped into three major lineages: one occupying the older eastern and central islands, another found mostly in the geographically distant northern islands, and a third distributed across the northern and younger western islands. However, this genetic structuring was not supported by nuclear markers, which did not show any clear sign of population differentiation among islands. Based on these results, nuclear gene flow was supposed to be high across the archipelago and is mainly the result of male-biased dispersal as males have been observed to swim to different islands during the breeding season [27]. This was one of the first examples of male-biased dispersal based on molecular evidence and is still used in textbooks (e.g. p. 229 in [28]).

In this study, we revisit the population genetic structure of marine iguanas in order to lend insight into the patterns of gene flow, genetic diversity, and demographic history of this species. Data is presented from over 1200 individuals from 23 populations sampled at two different time points using an increased number of nuclear markers (thirteen microsatellite loci) as well as the typically fast-evolving mtDNA control region (CR). We use this information to unravel patterns of past and current dispersal in marine iguanas, and discuss results within the context of the island progression hypothesis and sex-biased dispersal.

## Methods

### Sampling and genetic data collection

**Figure 1:**
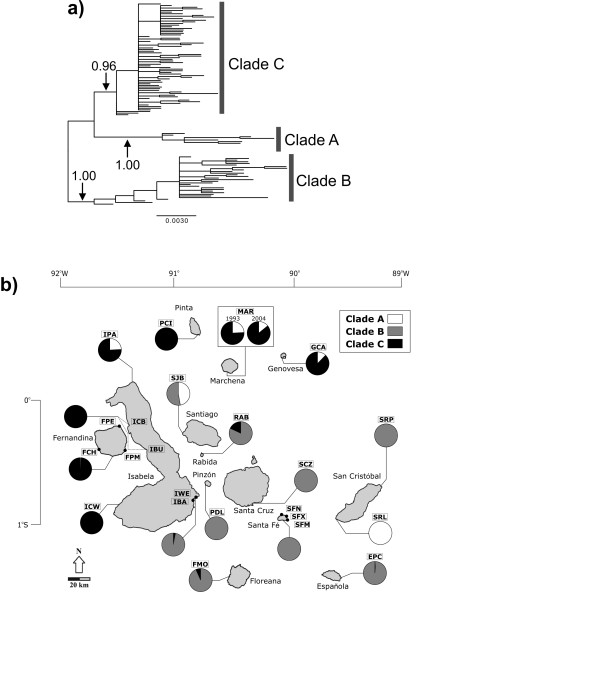
**Bayesian-based phylogeny of Galápagos marine iguanas and distribution of mitochondrial clades based on mtDNA CR data**. **a) **The topology and branch lengths were inferred using the program MRBAYES, but the rooting shown here was inferred using the software BEAST (see Methods). Branch lengths are in number of substitutions per site. Bayesian posterior probability values from MRBAYES are shown supporting the three major clades. **b) **Map of the Galápagos islands detailing population locations and symbols (details in Table 1). Pie charts illustrate the geographic distribution of the mtDNA CR clades resulting from the analysis of 1203 marine iguanas.

Marine iguanas were sampled during two different time periods, 1991/1993 and 2004. Samples from 1991/93 were a subset of those collected and analyzed from the Rassmann et al. study [27]. The total sampling effort resulted in more than 1200 marine iguana specimens spanning 13 islands and 23 populations (Figure 1b; Table 1). Eleven of these locations were sampled during both time periods to meet the objectives of a parallel study examining changes in genetic diversity due to an intense El Niño event [29].

**Table 1:** Information on Galápagos marine iguanas.

Island	Island Age (Myr)	Population size estimates	Population	*Microsatellite loci*				*Mitochondrial Control Region (CR)*						
				
				*N*	*N*_*alleles*_	H_obs_	k-test	N	H	Hd	*π*	*K*	F_s_	*D*
Española	3.31-3.54	1,700-21,000	EPC (Punta Cevallos)^*a*,*b*^	99	9.07	0.79	7 (p = 0.46)	98	6	0.656	0.00193	2.271	2.65	-1.20

Fernandina	0.04	15,000-120,000	FCH (Capo Hammond)^*a*^	28	9.5	0.72	9 (p = 0.11)	20	10	0.900	0.00281	3.311	-2.35	-0.80

			FPE (Punta Espinosa)^*a*,*b*^	99	11.6	0.78	8 (p = 0.25)	86	27	0.946	0.00332	3.911	-11.40**	-0.55

			FPM (Punta Mangle)^*a*,*b*^	78	10.7	0.79	10* (p = 0.037)	73	32	0.950	0.00328	3.872	-20.84**	-1.48*

Floreana	1.52-2.34	2,000-16,000	FMO (Punta Montura)^*a*,*b*^	51	8.7	0.78	6 (p = 0.67)	60	12	0.786	0.00423	4.983	0.98	0.18

Genovesa	0.35	900-15,000	GCA (Campamente)^*a*,*b*^	92	6.7	0.70	6(p = 0.67)	81	5	0.574	0.00299	3.021	6.26	0.48

Isabela	0.313-535	5,000-40,000	IBU (Bahia Urvina)^*b*^	17	8.9	0.79	8(p = 0.25)	15	10	0.924	0.00367	4.324	-2.65	-0.49

			IWE (Cabo West)^*b*^	-	-	-	-	13	5	0.628	0.00137	1.615	-0.58	-1.45

			ICB (Caleta Black)^*a*^	-	-	-	-	7	7	1.000	0.00364	4.286	-3.40*	-1.05

			ICW (Caleta Webb)^*a*,*b*^	40	10	0.74	8(p = 0.25)	30	8	0.724	0.00333	3.922	1.02	0.64

			IBA (Cerro Ballena)^*b*^	22	9.1	0.76	8(p = 0.25)	21	4	0.271	0.00078	0.914	-0.16	-1.72*

			IPA (Punta Albemarle)^*a*,*b*^	61	9.4	0.77	7(p = 0.46)	54	7	0.681	0.00337	3.969	3.42	0.62

Marchena	0.56	1,000-10,000	MBN 1993 (Bahia Negra)	30	7.7	0.76	5(p = 0.84)	29	4	0.685	0.00324	3.823	5.37	1.60

			MBN 2004 (Bahia Negra)	49	7.7	0.78	7(p = 0.46)	49	2	0.250	0.00212	2.500	8.57	0.33

Pinta	0.70	800-6,000	PCI (Caleta Ibetson)^*a*,*b*^	94	6.1	0.64	6(p = 0.67)	94	6	0.431	0.00118	1.388	0.63	0.42

Pinzón	1.40-1.73	200-900	PDL (Dumb Landing)^*a*^	9	5.9	0.76	7(p = 0.46)	12	4	0.682	0.00084	0.985	-0.65	-0.90

Rábida	1.06-1.56	200-2,000	RAB (No name)^*b*^	10	5.1	0.71	7(p = 0.46)	11	3	0.473	0.00308	3.756	4.00	-0.14

San Cristóbal	2.35-4.04	50-400	SRL (Loberia)^*a*,*b*^	82	6.3	0.70	4(p = 0.94)	83	3	0.616	0.00220	2.589	7.53	2.67

			SRP (Punta Pitt)^*a*^	20	3.9	0.51	5(p = 0.84)	20	1	0.000	0.00000	0.000	NA	NA

Santa Cruz	1.31-2.26	2,000-13,000	SCZ (Estacion/Camaño)^*a*,*b*^	135	9.5	0.80	7(p = 0.46)	116	2	0.017	0.00007	0.086	-0.56	-1.89

Santa Fé	2.85	3,000-16,000	SFN (North)^*a*^	52	8.4	0.80	8(p = 0.25)	52	4	0.587	0.00079	0.932	0.72	0.12

			SFM (Miedo)^*a*,*b*^	82	8.5	0.75	9(p = 0.11)	82	6	0.702	0.00283	3.341	4.53	2.18

			SFX (Bahia Paraiso)^*b*^	24	8.4	0.80	7(p = 0.46)	25	5	0.643	0.00175	2.067	1.14	0.43

Santiago	0.77-1.42	450-4,000	SJB (James Bay)^*a*,*b*^	51	7.7	0.76	10*(p = 0.037)	72	3	0.547	0.00607	7.160	18.49	3.81

Overall		37,000-280,000		1225				1203	106					

Population localities, sample sizes, island ages, and summary data for 13 microsatellite loci and the mitochondrial control region (CR).

Island ages are in millions of years (myr) and were derived from plate velocity and potassium-argon (KAr) dating estimates from multiple sources [3,17,82,86,87]. Estimates of population sizes were compiled in [78]. **Microsatellite loci data: ***N*, sample size for each population; *N*_*alleles*_, average number of alleles; *Hobs*, observed heterozygosity; intra-locus k-test statistic, number of loci out of 13 loci that showed negative k values and the corresponding p value (specific k values are provided by suppl. table 3). **Control Region data: ***N*, sample size for each population; *H*, number of haplotypes; *Hd*, haplotype diversity;, nucleotide diversity; *K*, average number of nucleotide differences between sequences; *F*_S_, Fu's neutrality statistic [51]; *D*, Tajima's neutrality statistic [52]. For the complete table: * and ** denote significance at the = 0.05 and = 0.01 levels respectively. Populations were sampled in 1991/1993^*a*^and/or 2004^*b*^.

Total genomic DNA was extracted from blood samples in a 96-well format using the QIAamp 96 DNA Blood Kit (QIAGEN Inc.) following the manufacturer's instructions. Complete mtDNA CR sequences (1183 bp) were generated for 1203 marine iguana specimens using PCR protocols that are described elsewhere [29,30]. We genotyped thirteen microsatellite loci for 1225 individuals: locus Am(GT)4 from [27] and twelve loci from [31] following the same procedures as described before [29].

### Phylogenetic analysis of CR sequences

CR sequences were edited in the program SEQUENCHER v4.2.2 [32] and aligned using the program MUSCLE v3.6 [33]. Unique haplotypes were defined and numbered using the program DNASP v4.20.2 [34]. The program MRMODELTEST v2 [35], which is based on code from the MODELTEST software [36], was used to evaluate the fit of different nucleotide substitution models to the data. The Hasegawa-Kishino-Yano model [37] with additional parameters for gamma distribution and fraction of invariable sites (HKY+G+I) provided the best fit to the haplotype data according to both the hierarchical likelihood ratio test and the Akaike information criterion. This substitution model was implemented in a Bayesian framework using the program MRBAYES v3.1.2 [38] with a search of 2.5 × 10^6 ^generations where the first 10% of parameter samples were discarded as burn-in.

Since the point of branch connection between marine iguana mtDNA phylogroups and Galápagos land iguanas has previously been shown to be unclear due to the large divergence between the two species [27], the root of the marine iguana phylogeny was inferred for CR sequences using a relaxed clock model implemented in the program BEAST v.1.4.7 [39]. As in the phylogenetic analysis, the HKY+G+I model of nucleotide substitution was applied. An evolutionary model was chosen where the substitution rates among branches were uncorrelated, and an expansion growth prior was assumed since marine iguanas exhibit shallow divergence and are likely still in the process of reaching population genetic equilibrium in parts of their range. The analysis was run twice with 2 × 10^6 ^generations each, and the first 10% of parameter values were discarded as burn-in. Adequate mixing was determined by examining the effective sample size and parameter trace values as visualized in the program TRACER v1.4 [40] and the two runs were combined to obtain an overall estimate of the posterior distribution of parameters.

### CR analysis of genetic diversity and population structure

The number of haplotypes (*h*), haplotype diversity (*Hd*), nucleotide diversity (*π*), and the average number of nucleotide differences between sequences (*K*) were calculated for each population using DNASP. Data were generated separately for the two temporal samples (1993 and 2004) from Marchena island, as a previous study showed that a bottleneck occurred in between samplings [29]. For all other populations, samples from the two time-points were combined. F_ST_ calculations based on Wright's *F*_ST_ [41] and an analysis of molecular variance (AMOVA) were performed in the program ARLEQUIN v3.11 [42] in order to determine the level of genetic differentiation within and between marine iguana populations and the following major island groups: north (Pinta, Marchena and Genovesa), west (Fernandina and Isabela), central (Santiago, Rábida, Pinzon, Santa Cruz, Floreana and Santa Fé) and east (Española and San Cristóbal).

We additionally tested for correlation of island age and molecular distance by applying a Mantel test as implemented in the program ARLEQUIN v3.11 [42]. For this *F*_ST _differentiation was estimated by haplotype frequencies between island populations and the corresponding age difference between islands was estimated as the difference of maximum island age as provided by Table 1. The significane of test results were determined by performing 10,000 randomizations.

### Population structure based on microsatellite loci data

Using microsatellite data, we employed a Bayesian-based analysis of population structure in order to identify genetic clusters, patterns of migration, and gene flow within *Amblyrhynchus *without using any *a priori *sampling information. Starting from individual genotypes, the number of genetic clusters was inferred using the program STRUCTURE v2.1 [43]. K (the number of inferred genetic clusters) ranged from 1 to 25 with 15 iterations for each K. The run length was set to 100,000 MCMC replicates after a burn-in period of one million replicates. For the ancestry model, we chose the admixture model with ALPHA being inferred from the data in combination with correlated allele frequencies. Based on the log probability of these runs, the true value of K was estimated using the approach of Evanno et al. [44]. The program DISTRUCT [45] was then used to graphically display the number of genetic clusters as well as genetic intermixing of individuals based on the true value of K in STRUCTURE. The program BAPS v3.2 [46], which uses a slightly different Bayesian approach than STRUCTURE to infer population differentiation, was also used to identify the optimal number of genetic clusters. The upper bound for the number of populations was set to the number of sampling locations in our dataset. This corresponded to 36 different sampling locations/events when considering different time points from the same location as independent sampling events. The optimal number of clusters identified by BAPS3.2 was selected from a list of the ten best visited partitions according to their log(ml) values (see [46]). Genotypic assignment of individuals to populations was tested by applying an individual-based self-assignment test using the program GENECLASS2 [47] and the implemented Bayesian approach of Rannala and Mountain [48].

In order to allow a direct comparison between our results and those of the Rassmann et al. study [27], we calculated overall differentiation as Reynolds *F*_ST _= -ln(1-Θ; [49]) using the program MICROSAT [50]. Analysis of molecular variance (AMOVA) was performed for the same sets of populations and major island groups as done for the equivalent mitochondrial analysis (see above) in the program ARLEQUIN v3.11 [42].

In order to determine the correlation between island age *vs *genetic distance (*F*_ST_) we used the same approach as described for the D-loop sequences (see above). The correlation between possible dispersal distance of marine iguanas *vs *genetic distance (*F*_ST_) was tested with a Mantel test as implemented in the program ARLEQUIN v3.11 [42]. The significane of test results were determined by performing 10,000 randomizations. Underlying dispersal distances in the matrix (see Table S1 [see Additional file 1]) were estimated under the assumption that iguanas from localities on different islands dispersed by drifting in the ocean, meaning that distances are the closest straight lines around intervening islands between localities. If localities were on the same island, the distances were the closest "coastal" distances - assuming that iguanas dispersed by either swimming or walking along the coast rather than walking across islands.

### Analysis of population expansion

Tests of recent population expansion were conducted on the combined population samples (i.e. 1991/93 and 2004 samples; see Table 1) for the mitochondrial and microsatellite datasets separately. For CR sequences, we obtained frequency distributions of pairwise nucleotide differences between sequences (mismatch distributions), Fu's *Fs *test [51], and Tajima's *D *statistic [52] using ARLEQUIN. Particular focus was placed on marine iguana populations on the comparatively young islands in the western (Fernandina and western Isabela) and northern (Genovesa, Marchena, and Pinta) regions of the archipelago, which may have been host to recent expansions in both range and population size. Details on methods and parameters used for these analyses are provided in the additional material [see Additional file 2].

The distribution of alleles at microsatellite loci were examined for signs of population expansion using the intra-locus *k *test as implemented in the Excel Macro KGTESTS [53]. Assuming a simple stepwise mutation model, locus-specific allele length distributions from constant-sized populations should have several modes, whereas a single mode is expected for expanding populations [54]. The intra-locus *k *test examines differences in allele length distributions for specific loci, where a negative k value is indicative of recent population expansion, as well as whether the number of negative k values across all loci is significant [55].

### Tests of sex-biased dispersal

Based on the results of the Rassmann et al. study [27] that indicated male-biased dispersal in marine iguanas, we performed four different tests of sex-biased dispersal for sets of adult individuals sampled in 1991/93 and 2004 [56]: i) The *F*_IS _statistic describes how well genotype frequencies follow expectations under Hardy-Weinberg Equilibrium and assumes that the dispersing sex will be a mixture of residents and immigrants resulting in a heterozygote deficiency and a positive *F*_IS _value; ii) *F*_ST _values should be lower for the dispersing sex because allele frequencies of the dispersing sex should be more similar across populations; iii) Immigrants tend to have a lower Assignment Index (AI; see [57] and [58] for details) than residents. Therefore, the dispersing sex should display, on average, a lower value of AI than the resident sex; iv) Since members of the dispersing sex will include both residents (with common genotypes) and immigrants (with rare genotypes), the variance of AI should be larger for the dispersing sex than for the resident sex. All tests were run in the program FSTAT version 2.9.3.2 [59] and the significance of different test results were determined by performing 10,000 randomizations.

## Results

### CR-based phylogenetic analysis and clade distribution

Complete mtDNA CR sequences were obtained for 1203 marine iguanas and contained 1183 bp, or 1179 bp when positions with insertions/deletions (indels) were removed as they did not provide significant information and would have extremly limited important applications within the program DNASP (the dataset with gaps can be obtained from SS or SG upon request). D-loop haplotypes have been deposited in GeneBank under accession numbers GQ293462-GQ293497 and EU278255-EU278326 and detailed information on haplotypes (e.g. island and clade designation) is provided by Table S2 [see Additional file 3]. The resulting alignment contained 106 haplotypes with a pairwise uncorrected sequence divergence of 1.6% between the most distant haplotypes. Thirty-five of these haplotypes were singletons.

**Figure 2:**
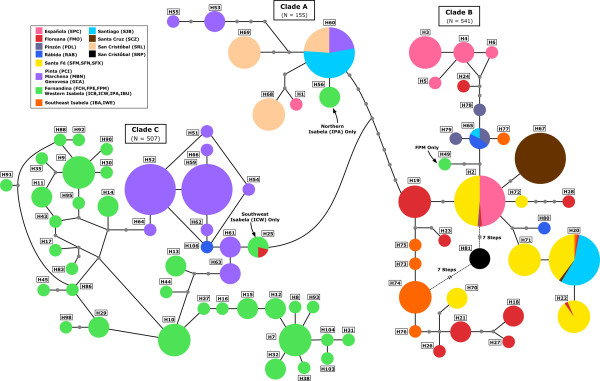
**Haplotype network constructed under statistical parsimony for all Galápagos marine iguana mtDNA CR haplotypes**. Haplotype numbers are preceded by the letter "H." The smallest circles denote missing haplotypes. The other four circle sizes reflect the number of individuals. In order of increasing area, this corresponds to 1-6, 7-20, 21-50, and 50 or more individuals. All singletons were removed from populations on Fernandina and western Isabela in order to more clearly display the overall phylogeographic patterns. Island distribution of each haplotype is indicated by color according to the embedded legend.

The topologies of trees generated by the MRBAYES (Figure 1a) and BEAST (data not shown) software were very similar and resulted in three general clades -designated A, B, and C - that were supported by high posterior probability values (PPV > 0.95). Under the relaxed clock model, Bayesian-based analysis in the software BEAST placed clade B as the basal group in the phylogeny. Many islands possessed haplotypes from multiple clades (Figure 1a and Figure 2). Clade A (PPV = 1) possessed the fewest haplotypes (*h *= 7) and was widely distributed across the archipelago; clade B (PPV = 1) contained 30 haplotypes that were found mostly on the central and eastern islands, but also on southern Isabela (IBA and IWE); clade C (PPV = 0.96) accounted for the majority of haplotypes (*h *= 69) and occurred on the northern (Pinta, Marchena, and Genovesa) and western (Fernandina and Isabela) islands as well as on the two central islands of Floreana and Rábida at low frequency. The average pairwise divergence within each of the three clades was low (~0.4%; Table S3a [see Additional file 4]) while the maximum divergence was apparently slightly higher in clade B (1.0%) than for the other two groups (0.8%). The average percent distance between clades ranged from 1.0 - 1.3% (Table S3b [see Additional file 4]).

### CR haplotype relationships and distribution

Statistical parsimony networks were constructed separately for each of the three clades. In clade A, haplotype H5 was the only variant that was widespread and found on multiple islands, including San Cristóbal to the southeast, Marchena to the north, and Santiago in the central archipelago (Figure 2). One clade A haplotype (H4) was also found on northern Isabela (IPA), showing that this lineage extends to the far west. In clade B, some haplotypes (e.g. H8, H15) were widespread across several of the eastern and central islands while others (e.g. H9-H12 on Española) formed groups that clustered within specific islands. Members of clade B were generally restricted to the eastern and central islands, except that the majority of southern Isabela haplotypes were also part of this clade. In addition, a clade B haplotype (H23) was identified from a single individual in the far western FPM population on Fernandina. Haplotypes from the island of Floreana appeared at several disjunct places in the network.

For clade C a high degree of reticulation was observed in the network. As a result, the complete network could not be represented in a clear fashion, and singleton individuals (N = 26) were removed from the statistical parsimony analysis to simplify the general phylogeographic patterns. The majority of clade C haplotypes were limited to the far western (Fernandina and western Isabela) and northern (Pinta, Marchena, and Genovesa) regions of the archipelago, although two clade C haplotypes, H60 and H106, were also found on two central islands, Floreana and Rábida.

### Population genetic diversity and differentiation based on CR data

Many populations from the west (i.e. Fernandina and western Isabela) showed high levels of genetic diversity (*Hd *> 0.900; Table 1). Conversely, a number of populations from the north, east, and central regions had particularly low levels of variation, many containing three haplotypes or less. The variation was particularly low on Santa Cruz, where 115 of the 116 individuals sampled shared the same CR haplotype.

The analysis of genetic structure (Table 2) showed that populations from the older central/eastern islands were highly differentiated from those in the north (*F*_ST_= 0.74) and west (*F*_ST_= 0.70). There was also significant structure between northern and western islands, but to a lesser degree (*F*_ST_= 0.42). Within regions, genetic distinctiveness among populations was much greater in the eastern/central archipelago (*F*_ST_= 0.55) than in the north (*F*_ST_= 0.18) or west (*F*_ST_= 0.09). However, some eastern/central islands (e.g. Santa Fé, Española) contained many individuals that shared haplotypes with populations from other islands (see Figure 1b). The most striking separation of marine iguana populations in the east/central region was observed on opposite ends of San Cristóbal island (*F*_ST_= 0.87); a result of the fact that haplotypes from these populations are from two different clades (Figure 1b; Figure 2). The results of the AMOVA, in which populations were grouped into northern, eastern/central, and western regions, showed that variation was similarly partitioned among groups (40.1%), among populations within groups (31.2%), and within populations (28.7%). These results were further supported by the fact that mitochondrial differentiation between island populations correlated with island age (r = 0.45; *p *= 0.004) and could explain 20% of the observed variation.

**Table 2:** Population structure of Galápagos marine iguanas.

	CR (*F*_ST_)	Microsatellite loci (*F*_ST_)
**Within Islands**		
Fernandina	0.00 ± 0.00	0.00 ± 0.00
Western Isabela	0.12 ± 0.07	0.03 ± 0.01
Santa Fé	0.16 ± 0.09	0.00 ± 0.00
San Cristóbal	0.87 ± 0.00	0.11 ± 0.09
		
**Within/Between**		
North	0.18 ± 0.08	0.13 ± 0.08
East/Central	0.55 ± 0.27	0.11 ± 0.05
West	0.09 ± 0.08	0.01 ± 0.01
North vs. East/Central	0.74 ± 0.12	0.14 ± 0.04
North vs. West	0.42 ± 0.19	0.12 ± 0.03
East/Central vs. West	0.70 ± 0.12	0.10 ± 0.02
		
**Among all 23**	0.58 ± 0.26	0.12 ± 0.05

Population structure of Galápagos marine iguanas within and between islands and major geographic regions based on CR data (*F*_ST _values) and 13 microsatellite loci (*F*_ST _values).

Only islands with multiple samples could be used to examine within-island differentiation. The two populations from southeastern Isabela (IBA and IWE) were included in the east/central since they show a higher genetic affinity to those islands. Standard deviations represent the mean over all pairwise comparisons.

### Microsatellite loci dependent population structure and gene flow

**Figure 3:**
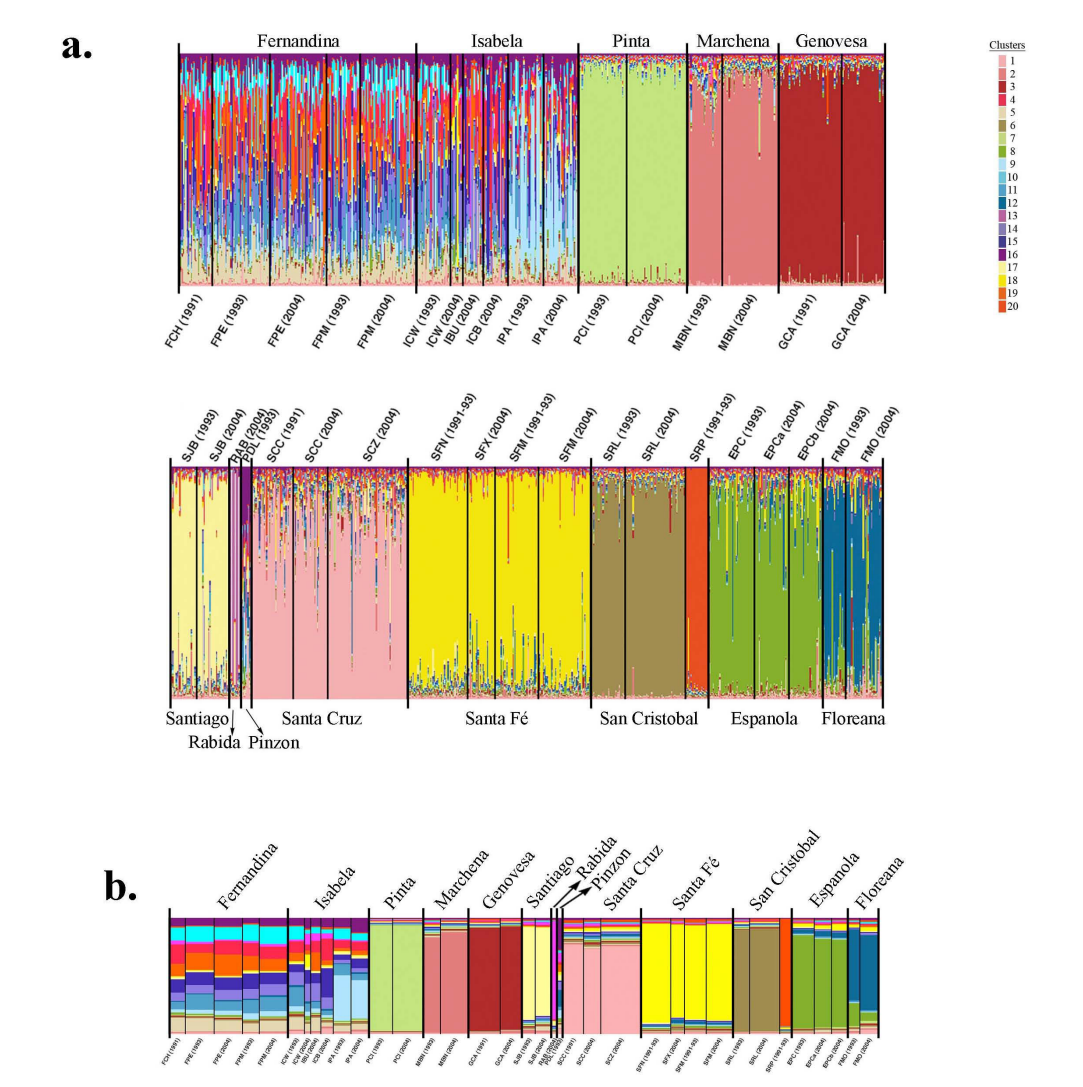
**Bayesian mixture analysis**. **a) **Individual-based mixture analysis of 1225 Galápagos marine iguanas to 20 genetic clusters as determined by the program STRUCTURE following the estimation of true number of genetic clusters (K) following [44] Evanno *et al*. (2005; see Figure S1 [see Additional file 5]). Note that individual samples taken at two different time points (i.e. in 1991/93 and 2004) on an island location represent the same genetic cluster. With the exception of Fernandina and Isabela all major islands each represent a distinct genetic cluster. **b) **Population-based mixture analysis for the same number of genetic clusters according to STRUCTURE. For (a) and (b) the program DISTRUCT [45] (Rosenberg 2004) was used to generate the display genetic clusters and degree of admixture of individuals.

Table 1 shows the average number of alleles and observed heterozygosity of each population that was genotyped for thirteen microsatellite loci. Complete genotypic data was obtained for 1225 individuals from 23 sampling locations as well as from different time points for the same island (see Table 1 for details). Applying the approach of Evanno et al. [44], the most likely number of genetic clusters for the complete dataset based on the results of STRUCTURE was estimated at 20 (see Figure 3 and Figure S1 [see Additional file 5]). The individual-based Bayesian structure analysis using BAPS indicated the same number of genetic clusters (see Table S4 [see Additional file 6]).

We found different patterns of genetic differentiation across the archipelago. Individuals sampled at different time points (i.e. 1991/93 and 2004) from the northern (Pinta, Marchena, Genovesa), central (Santiago, Santa Fé, Santa Cruz) and one of the eastern (Española) islands each form a single island-specific genetic cluster with a low degree of intermixing with genetic clusters from other islands. On the other easternmost island of San Cristóbal, two highly distinct genetic clusters were found corresponding to the western (SRL) and eastern (SRP) populations. The strong genetic differentiation among these islands (as well as among populations within islands in the case of San Cristóbal) was further supported by the results of the individual-based self-assignment test. Proportions of individuals correctly assigned to these island populations ranged from 96-100% indicating low levels of gene flow between islands (Table 3). Gene flow from Española to Floreana was indicated by 12% of individuals sampled on Floreana, but incorrectly assigning to Española. The smaller islands such as Pinzon and Rábida show signatures of genetic exchange with the larger islands of Fernandina and Isabela/Santiago, respectively (see Table 3).

**Table 3:** Results of the self-assignment test.

Island population	Proportion of correctly assigned individuals	Proportion of non-correctly assigned individuals
Fernandina	0.82	0.18 (Isabela)
San Christobal (SCR)	1.0	-
San Christobal (SRP)	1.0	-
Floreana	0.82	0.12 (Española)
		0.06 (Santa Cruz)
Genovesa	1.0	-
Marchena	1.0	-
Pinta	1.0	-
Santiago	0.96	0.04 (Isabela)
Santa Cruz	0.98	0.02 (Isabela)
Española	0.96	0.04 (Floreana)
Isabela	0.66	0.3 (Fernandina)
		0.007 (Floreana)
		0.007 (Santa Cruz)
		0.007 (Santa Fé)
Rábida	0.8	0.1 (Isabela)
		0.1 (Santiago)
Pinzon	0.78	0.22 (Fernandina)
Santa Fé	0.994	0.006 (Isabela)

Results of the self-assignment test of 1225 Galápagos marine iguana samples using the approach of Rannala and Mountain [48] as implemented in the program GENECLASS2 [47]. Proportion of "correctly" assigned individuals includes individuals that were assigned to their island of origin irrespective of the sampling period (i.e. sampled in 1991/93 or 2004; see Material and Methods for details), whereas the second column gives the proportion of individuals assigned to islands other than the island of origin.

In contrast, STRUCTURE analysis (Figure 3a) showed that individuals sampled on Fernandina and Isabela are genetically similar, with populations on both islands showing a high degree of genetic mixing. The population from Punta Albermarle (IPA) in northern Isabela was an exception since it was genetically differentiated from the remaining populations on Fernandina and Isabela. Also, the self-assignment analysis indicated high levels of gene flow between these two islands as all of the incorrectly assigned individuals on Fernandina (18%) were from Isabela and nearly all incorrectly assigned individuals on Isabela (30%) were from Fernandina (Table 3). Importantly, the nine distinct genetic clusters recovered by our analyses on Fernandina and Isabela are mostly absent from the remaining islands of the archipelago (Figure 3b).

The average *F*_ST _values between the 16 populations sampled in 1991/93 and the 18 populations sampled in 2004 were quite similar (0.13 ± 0.057 and to 0.12 ± 0.051, mean ± SD, respectively). Within-island *F*_ST _differentiation ranged from 0 (on Fernandina) to 0.11 (on San Cristóbal) and *F*_ST _between islands from the same region ranged from 0.01 (west) to 0.13 (north). Differentiation between geographic regions was low, ranging from 0.1 (East/Central vs. West) to 0.14 (North vs. East/Central) (see Table 2). In line with these results, the AMOVA analysis revealed that only 3% of the variation was partitioned between geographic groups, 8% among populations within geographic groups, and 89% within populations. Also, no evidence for a relationship between island age and genetic distance was found on the basis of microsatellite loci (r = 0.14; *p *= 0.25). A slight (r = 0.26), but highly significantly (*p *= 0.001) correlation between possible dispersal distances and genetic distance was found that could explain 7% of the observed variation between matrices (see also Figure S2 [see Additional file 7] for corresponding scatter plots).

### Analysis of population expansion

For the mtDNA dataset, both the western and northern populations conformed to the model of sudden-expansion (*p *> 0.05; Figure S3a,b [see Additional file 8]), which was further supported by low raggedness values (*p *> 0.05). However, only the west showed a clear unimodal mismatch pattern which is suggestive of recent population expansion (Figure S3a-d [see Additional file 8]). Also, highly negative *F*_S _values (*p *< 0.05) were obtained for two sites on Fernandina (FPE and FPM) and for one site on western Isabela (ICB), indicating an excess of recent mutations in these populations due to population growth (see Table 1). Of these three sites, only FPM was also significant for Tajima's *D *neutrality test (*p *= 0.04), but with a much weaker signal than for the *F*_S _test. In addition, when all the western populations were combined, *F*_S _values were negative and significant (*F*_S _= -25.729; *p *= 0.00), but *D *values were not (*D *= -1.144; *p *= 0.11).

Intra-locus *k*-test statistics based on microsatellites showed evidence of significant recent population expansion for only two populations. Ten out of thirteen loci (Table 1 and Table S5 [see Additional file 9]) showed negative *k *values that are indicative of recent population expansion for the FPM site on Fernandina and the Saint James Bay (SJB) site on Santiago.

### Analysis of sex-biased dispersal

Table 4 provides the results of the four different test statistics for sex-biased dispersal for marine iguana individuals sampled in 1991/93 and 2004. Although some of the test results are slightly different for females and males sampled in 1991/93 and 2004, none showed significant evidence of detectable genetic consequences from potentially sex-biased dispersal in marine iguanas.

**Table 4:** Test for sex-biased dispersal in Galápagos marine iguanas.

Sampling period	*F*_IS _statistic	*F*_ST _statistic	Mean AIc test	Variance AIc test
1991/93	F = 0.023	F = 0.117	F = 0.124	F = 11.24
	M = 0.040	M = 0.115	M = -0.106	M = 12.07
	p = 0.11	p = 0.35	p = 0.22	p = 0.35
				
2004	F = 0.033	F = 0.106	F = -0.145	F = 14.49
	M = 0.007	M = 0.129	M = 0.136	M = 13.39
	p = 0.98	p = 0.82	p = 0.78	p = 0.77

The results of four tests (*F*_IS _test, *F*_ST _test, corrected Mean Assignment Index test and variance of corrected assignment test; see material and methods and [56] for details) are shown for females (F) and males (M) as sampled in 1991/93 and 2004. Note that none of the tests for both sampling periods provide significant differences (p-value) concerning dispersal of sexes.

## Discussion

### Do marine iguanas follow the progression hypothesis?

Genetic studies have revealed that many species in the Galápagos follow the progression hypothesis, where islands are colonized in order of their emergence (reviewed in [2]). This pattern has been attributed to the relative ease in which migrating individuals can successfully occupy younger, uninhabited islands in comparison to older ones, where populations have already been established [18]. The progression hypothesis appears most supported by poorly dispersing species such as giant tortoises [60,61], lava lizards [62,63], land snails [7], and Galápagos land iguanas [64,65] since the probability of back migration to older islands is low. Conversely, the phylogeographic patterns of more mobile organisms such as Darwin finches ([1]; but see [4]), insects (e.g. weevils; [10]), sea lions [66] and hawks [67] do not always conform to the geologic history of the islands. In the case of Galápagos sea lions and hawks genetic data suggest that population structure was established and affected by quite different factors after a rapid population expansion across the whole archipelago: sea lions diverged according to ecological differences in a western and eastern clade [66], whereas an island-dependent population structure due to limited dispersal between islands was found for the Galápagos hawks [67].

Galápagos marine iguanas feed exclusively on aquatic algae and are closely tied to the coastal environment throughout their lives [20,68,69]. Large individuals are even known to forage offshore in the subtidal zone, swimming up to several hundred meters to reach feeding sites [20]. It is therefore reasonable to expect that these animals can disperse easily - either actively or passively - and that their evolutionary history should not conform to the progression hypothesis.

On a broad scale, the current study provides evidence that the progression hypothesis appears to hold true for marine iguanas. Our CR analyses, as well as the previously published cyt*b *study [27], suggest that clade B may be basal to clades A and C (Figure 1a). Biogeographically, this makes sense since clade B is found almost exclusively on the eastern and central islands (Figure 1b) that are considered to be the oldest in the archipelago. Meanwhile, the majority of clade C haplotypes was identified on younger islands in the north and west. The basal position of clade B would be made stronger if Galápagos land iguanas - the acknowledged sister species of marine iguanas -could be used as an outgroup for phylogenetic analysis. However, when this was attempted using Bayesian-based phylogenetic inference, independent runs failed to converge after 1 × 10^7 ^generations. This outcome is likely due to the large divergence between marine and land iguana CR sequences (approximately 10%) compared to the low divergence within marine iguanas (max. 1.6%), reflecting the long timeframe in which the two species have independently evolved on both existing and submerged islands.

Due to the difficulty in rooting the marine iguana phylogeny with CR data, frequency-based genetic information provides important additional support for the progression hypothesis at the large scale. The genetic division between populations from the eastern/central archipelago and those from the northern and western islands was evident in the CR haplotype frequencies, which showed high levels of differentiation as expressed by rather high *F*_ST_-values between regions (see Table 2). *F*_ST _differentiation based on microsatellite loci for corresponding comparisons is much lower (Table 2) and not in line with the mitochondrial data.

One consequence of the progression hypothesis is that levels of genetic structure are higher among populations on older islands since these are more likely to be at equilibrium and to have undergone significant lineage sorting. For example, in Galápagos tortoises, mitochondrial haplotypes are largely endemic to specific islands in the eastern archipelago while those in the west are often shared between populations and depict an intricate history of genetic exchange [60,61]. A qualitatively similar pattern is also seen for marine iguanas. Mitochondrial CR differentiation was much higher between populations from the older eastern/central archipelago than among the younger western and northern islands. Moreover, levels of genetic differentiation among populations on the youngest (~35,000-535,000 years) and westernmost islands of Fernandina and Isabela were not significantly different from 0 (see Table 2).

There are some exceptions to the general east/west division of marine iguanas in the archipelago that are evident from the CR data. While clade B haplotypes did predominate in the eastern/central region in our study, they were also in the majority on southeastern Isabela, suggesting that this part of the island is biogeographically more connected to the east/central archipelago than to the west. In addition, a single clade B haplotype (H23) was also found on the far-western island of Fernandina, demonstrating the potential for rare long-distance dispersal from the east. Similarly, haplotypes from clade C, which were previously shown to be restricted to the north and west, were also identified on two of the central islands, Floreana and Rábida. A unique clade A haplotype was also identified on northern Isabela, showing that this lineage extends across the entire northern archipelago. The ancestral haplotype in this clade (H5) was widely distributed, existing on San Cristóbal to the east, and Santiago and Marchena in the north-central archipelago. Since San Cristóbal is one of the two oldest islands in the Galápagos, clade A likely radiated from there to the north and west, all the way to the northern tip of Isabela. This fits well with the patterns of island age as well as the path of the prevailing Humboldt current which approaches the archipelago from a southeasterly direction [70] and is thought to have produced similar east to west colonization patterns in other Galápagos organisms [2,63].

While the progression hypothesis generally explains the overall east/west division of marine iguanas, dispersal patterns have produced a more complicated history within regions. Unlike tortoises and other Galápagos organisms that exhibit complete genetic structuring among older populations, many marine iguana CR haplotypes are shared between different islands in the eastern/central region (Figure 1b and Figure 2). For example, more than half of the marine iguanas sampled on Santa Fé have CR haplotypes in common with individuals from other islands. In addition, the statistical parsimony analysis (Figure 2) shows that haplotypes restricted to several of the older islands in the eastern archipelago (e.g. Española and Santa Fé) are connected to other eastern/central island haplotypes via haplotypes from younger central islands (e.g. Pinzón, Isabela), suggesting that migration routes have not proceeded strictly according to the age of island emergence. The appearance of Floreana haplotypes throughout the network shows that this island has been subject to continual genetic exchange with other islands.

### Microsatellite loci based population structure - evidence for sex-biased dispersal in marine iguanas?

Bayesian cluster analysis of microsatellite loci genotypes shows that most marine iguanas belong to highly distinct genetic island clusters (Figure 3). This result was independently supported by the high assignment rate of individuals to their island of origin for the majority of islands (Table 3). The Bayesian cluster analysis is especially convincing for two reasons. First, individuals sampled at different time points (i.e. in 1991/93 and 2004) but at the same sampling location were grouped together as one genetic unit. Second, the same number of genetic clusters was obtained by two different Bayesian clustering methods (STRUCTURE and BAPS). The microsatellite loci data therefore suggest that the structuring of marine iguana populations generally follows a pattern of one genetic unit per island. A similar pattern was also recently demonstrated for Galápagos lava lizards from the genus *Microlophus *[71].

Based on *F*_ST_analyses of nuclear genetic data, high levels of gene flow between islands and populations were reported [27]. The overall *F*_ST_ was approximately 0.1 for the 22 marine iguana populations examined. Although the average *F*_ST_ differentiation found in our study was in the same range (*F*_ST_= 0.13 and *F*_ST_= 0.12, respectively, for 1991/93 and 2004 samplings), the high level of genetic differentiation between islands indicated by Bayesian cluster analyses suggests that recent gene flow has been limited, and consequently that *F*_ST_ values may provide little information about fine scale population structure (see reviews [72,73]). Another issue of microsatellite-based *F*_ST_ estimates is the underestimation of genetic structure due to allele size homoplasy. Such a pattern may result from the characteristically high mutation rates and allelic polymorphism of microsatellite loci and may diminish the observed genetic distance (reviewed in [74]). As an example, when microsatellite loci and sequences of the nuclear coded internal transcribed spacer (ITS-1) were analysed in the coral species *Corallium rubrum *across the western Mediterranean, ITS-1 sequences revealed significant genetic structure between different geographic regions while microsatellite loci failed to show any correlation between *F*_ST_-based estimates for geographic distances ranging from 30-2650 km [75]. In another study in the same organism, however, very fine scale structuring based on microsatellites was observed for distances of ten meters to one km [76] indicating that spatial genetic structure does exists in this system but may not be detectable using microsatellite-based *F*_ST_ estimates over large distances and/or time frames.

Since the study of Rassmann et al. found significant differentiation between islands and regions based on maternally inherited mtDNA markers but not on bi-parentally inherited nuclear microsatellite and minisatellite loci, they concluded that male-mediated dispersal and gene flow was likely responsible for this discrepancy [27]. Support for this hypothesis came from frequent field observations in which male marine iguanas have been observed to swim to different islands during the breeding season - apparently to establish breeding territories (e.g. Santa Cruz and Caamaño; [77]). After the breeding season they return to the larger island where they "live". On the other hand females have been observed swimming to some islands (e.g. Plaza Sur, Plaza Norte and "Devine's Bay" in Academy Bay) during the breeding season and returned to their resident island after oviposition (unpublished data).

In our study none of the four tests conducted showed significant evidence for sex-biased dispersal in marine iguanas (Table 4). Since the set of individuals analyzed in the Rassman et al. study [27] for three microsatellite loci corresponds to the 1991/93 samples analyzed for thirteen microsatellite loci in our study, it was possible to directly compare of *F*_ST_-based test statistics between the two studies. In the former study [27] males displayed a significantly lower *F*_ST_-value (*F*_ST_= 0.09) than females (*F*_ST_= 0.12) suggesting that males disperse more than females. Conversely, *F*_ST _ estimates for the same set of males and females, which were genotyped at thirteen microsatellite loci, were not significantly different (males, *F*_ST_ = 0.115; females, *F*_ST_= 0.117; p = 0.35; Table 4). For individuals sampled in 2004, we even found higher *F*_ST_ values for males (*F*_ST_= 0.129) than for females (*F*_ST_= 0.106), but also non-significant (*p *= 0.82). These findings suggest that the results of the Rassman et al. study [27], which were based only on three microsatellite loci, should be approached cautiously. Since sex bias has to be intense to be detected by any of the applied test statistics [56], we cannot rule out that such a bias might exist in the case of single dispersers. However, we can exclude that this is a major demographic characteristic of marine iguana populations, as previously suggested.

Beyond the results of the sex-biased dispersal tests, the observed genetic patterns make high dispersal rates in marine iguanas unlikely. Bayesian-based population structure analysis (Figure 3) indicates that, except in a few cases (e.g. Isabela and Fernandina), recent migration and gene flow between islands is low. The fact that we recovered separate genetic units in the west (location SRL) and east (location SRP) on San Cristóbal (also supported by the mitochondrial data; see Figure 1b; Figure 2) as well as detected population structure between populations on Isabela (IPA *versus *the rest of the Isabela; see Figure 3b) indicates that gene flow of marine iguanas can be limited even along the coasts of the same island. These findings are not in line with expectations of high male-mediated gene flow [27].

### Evolution of high genetic diversity on Isabela and Fernandina

Marine iguana populations on the westernmost islands of Isabela and Fernandina are among the largest in the Galápagos, with Fernandina alone numbering in the hundreds of thousands [78]. Individuals from these populations are also the largest in the archipelago with a maximum body mass of 12 kg for individuals in southwestern Isabela [79]. Both large population size and individual body size have been attributed to the high marine productivity generated by upwelling of the nutrient-rich Cromwell current along the western archipelago [19,80]. The same region also harbors nearly 95% of the overall Galápagos penguin (*Spheniscus mendiculus*) population [81].

The western marine iguana populations also possess a high level of genetic diversity for both mitochondrial and microsatellite data, even though they occur on the two most recently emerged islands. The emergence age of the oldest volcano on Isabela is less than 535,000 years, while the emergence age of Fernandina is estimated to be only 35,000 years [82]. In contrast, central and southern islands of the archipelago are 1-4 million years old. Sixty-seven out of the total 106 mitochondrial control region haploytypes are found on Fernandina and Isabela with 65 haplotypes being distinct to these islands (Figure 1 and Figure 2) resulting in high levels of haplotype diversity. In contrast populations on the other islands showed significantly fewer haplotypes and lower levels of haplotype diversity. Also, at the level of microsatellite loci, up to nine different genetic clusters were detected for Fernandina and Isabela (Figure 3b), whereas a single predominant cluster is found in populations from most other islands. These genetic clusters are unique to the Isabela and Fernandina populations and therefore must have recently evolved on these islands.

On a broad scale across all islands mitochondrial as well as nuclear diversity indices did not correlate with island size (i.e. island perimeter; (see Figure S4 [see Additional file 10]). The most obvious explanation for the higher level of genetic diversity on the younger islands is that large population sizes along with recent population growth have resulted in many new mutations combined with decreased effects of genetic drift. Indeed, western populations on Isabela and Fernandina show clear signs of sudden-expansion based on mtDNA data as evidenced by a clear unimodal mismatch pattern (Figure S3 [see Additional file 8]) and highly negative *F*_S _values for Punta Espinosa and Punta Mangle on Fernandina (see Table 1) indicating an excess of recent mutations. The microsatellite loci-based *k*-test statistic showed significant support for recent population expansion for the Punta Mangle population on Fernandina (Table 1 and Table S5 [see Additional file 9]), but not for any of the other western populations.

However, the analysis of CR haplotypes also shows that western marine iguana populations possess mtDNA haplotypes typical of some central, northern, and southern populations. This variation could have been introduced into the western populations through founder events from multiple sources. An increased variation within populations due to multiple recent colonizations has been shown for the invasive brown anolis (*Anolis sagrei*; [83]. In this respect, the finding that mitochondrial CR haplotypes of marine iguanas from southern Isabela are connected to haploytypes typical of the central islands (Santa Cruz and Floreana) - a similar picture that is seen also in Galápagos tortoises [60,84,85] - underpins the colonization of Isabela from multiple sources. In the land iguana *C. subcristatus*, mitochondrial DNA sequence data indicate a single founder event of western islands from a single source located in the central islands [65]. Subsequently *C. subcristatus *seems to have dispersed in a south to north direction on Isabela. However, the occurrence of an old separate lineage on Isabela (now recognized as a separate species; [65]) indicates that land iguanas have colonized Isabela at least twice.

## Conclusions

This comprehensive analysis of the population structure of Galápagos marine iguanas both supports and transforms our previous knowledge about the microevolution of this unique species. The detailed mtDNA analyses trace back migration routes in the evolutionary past and suggest that colonization of islands progressed from geologically older to younger islands in the archipelago. The existence of highly differentiated genetic clusters among islands as well as no specific support for sex-biased dispersal conflict with the previously held view of high male gene flow among islands. More generally, our study demonstrates how the development of recent analytical tools such as Bayesian clustering analysis and DNA sequence-based demographic analyses allow us to tease apart the role of past and present gene flow in shaping current patterns of population differentiation.

## Authors' contributions

Project conception and design: SS SG AC HLS. Performed laboratory work: SS SG DL TCH. Data analysis: SS SG DL TCH. Wrote the paper: SS SG AC MR. Figure design: DL SG SS. Field sampling: SS SG AC MR CM SR HMS GG GO AMP. All authors read and approved the final manuscript.

## Supplementary Material

Additional file 1 **Table S1: Possible dispersal distances of marine iguanas**. Click here for file

Additional file 2 **Supporting information on mitochondrial CR analyses **[88-93]. Click here for file

Additional file 3 Table S2: Detailed infromation on D-loop haplotypes. Click here for file

Additional file 4 **Table S3: General characteristics of the three mitochondrial clades of Galápagos marine iguanas**. Click here for file

Additional file 5 Figure S1: Estimation of genetic clusters according to Evanno et al. Click here for file

Additional file 6 Table S4: Best found partitions according to BAPS v3.2. Click here for file

Additional file 7 Figure S2: Scatter plot *F*_ST_(based on microsatellite loci) versus possible dispersal distance. Click here for file

Additional file 8 Figure S3: Mismatch distribution of Galápagos marine iguana D-loop haplotypes. Click here for file

Additional file 9 Table S5: Intra locus specific k-values of thirteen microsatellite loci of Galápagos marine iguana populations. Click here for file

Additional file 10 **Figure S4: Scatter plot with regression analysis of island perimeter as measured by NOAA GIS resource (World Vector Coastline) *vs *genetic diversity values**. Click here for file
