# Supplementary material for: Progressive colonization and restricted gene flow shape island-dependent population structure in Galápagos marine iguanas (Amblyrhynchus cristatus)
Source: BMC Evol Biol. 2009 Dec 22;9:297. doi: 10.1186/1471-2148-9-297 (PMC2807874; doi:10.1186/1471-2148-9-297)
Supplement: Additional file 2 — Supporting information on mitochondrial CR analyses [88-93]. [file 1471-2148-9-297-S2.DOC]

**Supporting information on mitochondrial CR analyses**

*Test for nuclear insertions of mtDNA (i.e. NUMTs)*

To investigate whether sequences represented nuclear insertions of mtDNA (i.e. NUMTs), we also amplified a long fragment (~4 kb) spanning most of cyt*b*, the entire CR and 12S ribosomal RNA (rRNA), and a portion of the 16S rRNA (long-mtDNA), from nine randomly chosen individuals from different populations. Given that the majority of NUMTs are less than 1 kbp in length **[**88,89**]**, an exact match between the CR-only and the long-mtDNA fragments would support the mitochondrial origin of the CR data. The long (~4kb) mtDNA PCR fragment amplified was identical to the shorter CR fragments from the same individuals in the area of overlap. In addition, the long mtDNA sequences were also identical or nearly identical to published cytb sequences **[**27**]**.

*CR-based analysis of population expansion*

Frequency distributions of pairwise nucleotide differences between sequences (mismatch distributions) were constructed in ARLEQUIN for Galápagos marine iguanas from the western (Fernandina and western Isabela) and northern (Genovesa, Marchena, and Pinta) regions of the archipelago. These islands are comparatively young (Table 1) and may have been host to a recent expansion in both range and population size. Mismatch distributions are often unimodal in populations that have undergone a recent demographic expansion because most of the coalescent events occur during the initial burst of population growth **[**90**]**. A non-linear least squares approach was used to find the values of the demographic parameters (τ, expansion time; *θ*_0_; *θ*_1_) which minimize the sum of squares deviation (*SSD*) between the observed mismatch distribution and the expected distribution under the stepwise expansion model **[**91**]**. This model was extended to account for heterogeneity in mutation rates which are characteristic of mtDNA control region data **[**92**]**. The parameter values of the expected theoretical distribution were used to simulate 10,000 sample distributions using a parametric bootstrap approach **[**92**]**. These simulations provide confidence intervals for the expected distribution as well as the basis for a test of fit of the observed distribution to the stepwise expansion model. The model is rejected if 95% or more of the simulated distributions have a lower *SSD* than the observed distribution when fitted to the expected curve. The raggedness index **[**93**]**, which measures the magnitude of change in the frequency of pairwise differences, was also tested with the same bootstrap approach.

Fu **[**51**]** (1997) showed that the *F_S_* test is particularly powerful for detecting population growth. In this test, an excess of rare alleles results in negative values of *F_S_* and reflects the occurrence of new mutations due to a recent increase in population size. Tajima’s *D* statistic is another popular method of detecting population expansion and takes on significantly negative values when the number of segregating sites (*S*) is much larger than *θ*, as estimated by the average number of pairwise differences between sequences. This occurs when population growth leads to many new mutations, resulting in an increase in *S* with little influence on *θ*. In order to obtain a test statistic for *F*_S_ and *D*, *θ* estimates from each population were used to simulate 5000 samples using ARLEQUIN. The locations of the observed values on the simulated distribution define critical points for rejecting the null hypothesis of no expansion.
