# Supplementary material for: Progressive colonization and restricted gene flow shape island-dependent population structure in Galápagos marine iguanas (Amblyrhynchus cristatus)
Source: BMC Evol Biol. 2009 Dec 22;9:297. doi: 10.1186/1471-2148-9-297 (PMC2807874; doi:10.1186/1471-2148-9-297)
Supplement: Additional file 3 — Table S2: Detailed infromation on D-loop haplotypes. [file 1471-2148-9-297-S3.PDF]

GenBank Number	Haplotype	Clade	EPC	FCH	FPE	FMO	FPM	GCA	IPA	IBU	IBA	ICB	IWE	ICW	MAR'93	MAR'04	PCI	PDL	RAB	SJB	SCZ	SRL	SFM	SFN	SFX	SRP	Total
EU278255	1	A	1	0	0	0	0	0	0	0	0	0	0	0	0	0	0	0	0	0	0	0	0	0	0	0	1
EU278256	2	B	51	0	0	2	0	0	0	0	0	0	0	0	0	0	0	1	0	0	0	0	31	7	13	0	105
EU278257	3	B	21	0	0	0	0	0	0	0	0	0	0	0	0	0	0	0	0	0	0	0	0	0	0	0	21
EU278258	4	B	17	0	0	0	0	0	0	0	0	0	0	0	0	0	0	0	0	0	0	0	0	0	0	0	17
EU278259	5	B	4	0	0	0	0	0	0	0	0	0	0	0	0	0	0	0	0	0	0	0	0	0	0	0	4
EU278260	6	B	4	0	0	0	0	0	0	0	0	0	0	0	0	0	0	0	0	0	0	0	0	0	0	0	4
EU278261	7	C	0	3	9	0	3	0	4	1	0	1	0	0	0	0	0	0	0	0	0	0	0	0	0	0	21
EU278262	8	C	0	1	0	0	1	0	0	0	0	0	0	0	0	0	0	0	0	0	0	0	0	0	0	0	2
EU278263	9	C	0	5	8	0	11	0	0	2	0	1	0	4	0	0	0	0	0	0	0	0	0	0	0	0	31
EU278264	10	C	0	3	10	0	6	0	27	1	0	0	0	0	0	0	0	0	0	0	0	0	0	0	0	0	47
EU278265	11	C	0	3	6	0	8	0	0	0	0	0	0	0	0	0	0	0	0	0	0	0	0	0	0	0	17
EU278266	12	C	0	0	6	0	2	0	1	0	0	0	0	0	0	0	0	0	0	0	0	0	0	0	0	0	9
EU278267	13	C	0	1	6	0	2	0	0	0	0	0	0	0	0	0	0	0	0	0	0	0	0	0	0	0	9
EU278268	14	C	0	0	1	0	5	0	0	0	0	1	0	0	0	0	0	0	0	0	0	0	0	0	0	0	7
EU278269	15	C	0	1	4	0	3	0	0	1	0	1	0	0	0	0	0	0	0	0	0	0	0	0	0	0	10
EU278270	16	C	0	0	2	0	1	0	0	0	0	0	0	0	0	0	0	0	0	0	0	0	0	0	0	0	3
EU278271	17	C	0	0	1	0	1	0	0	0	0	0	0	0	0	0	0	0	0	0	0	0	0	0	0	0	2
EU278272	18	B	0	0	0	9	0	0	0	0	0	0	0	0	0	0	0	0	0	0	0	0	0	0	0	0	9
EU278273	19	B	0	0	0	25	0	0	0	0	0	0	0	0	0	0	0	0	0	0	0	0	0	0	0	0	25
EU278274	20	B	0	0	0	1	0	0	0	0	0	0	1	0	0	0	0	0	0	35	1	0	3	14	8	0	63
EU278275	21	B	0	0	0	8	0	0	0	0	0	0	0	0	0	0	0	0	0	0	0	0	0	0	0	0	8
EU278276	22	B	0	0	0	1	0	0	0	0	0	0	0	0	0	0	0	0	0	0	0	0	27	1	2	0	31
EU278277	23	B	0	0	0	2	0	0	0	0	0	0	0	0	0	0	0	0	0	0	0	0	0	0	0	0	2
EU278278	24	B	0	0	0	1	0	0	0	0	0	0	0	0	0	0	0	0	0	0	0	0	0	0	0	0	1
EU278279	25	C	0	0	0	4	0	0	0	0	0	0	0	15	0	0	0	0	0	0	0	0	0	0	0	0	19
EU278280	26	B	0	0	0	4	0	0	0	0	0	0	0	0	0	0	0	0	0	0	0	0	0	0	0	0	4
EU278281	27	B	0	0	0	2	0	0	0	0	0	0	0	0	0	0	0	0	0	0	0	0	0	0	0	0	2
EU278282	28	B	0	0	0	1	0	0	0	0	0	0	0	0	0	0	0	0	0	0	0	0	0	0	0	0	1
EU278283	29	C	0	0	1	0	0	0	7	0	0	0	0	0	0	0	0	0	0	0	0	0	0	0	0	0	8
EU278284	30	C	0	0	1	0	1	0	0	0	0	0	0	0	0	0	0	0	0	0	0	0	0	0	0	0	2
EU278285	31	C	0	0	1	0	1	0	0	0	0	0	0	0	0	0	0	0	0	0	0	0	0	0	0	0	2
EU278286	32	C	0	0	0	0	2	0	0	0	0	0	0	0	0	0	0	0	0	0	0	0	0	0	0	0	2
EU278287	33	C	0	0	0	0	1	0	0	0	0	0	0	0	0	0	0	0	0	0	0	0	0	0	0	0	1
EU278288	34	C	0	0	0	0	1	0	0	0	0	0	0	0	0	0	0	0	0	0	0	0	0	0	0	0	1
EU278289	35	C	0	0	0	0	3	0	0	0	0	0	0	0	0	0	0	0	0	0	0	0	0	0	0	0	3
EU278290	36	C	0	0	0	0	1	0	0	0	0	0	0	0	0	0	0	0	0	0	0	0	0	0	0	0	1
EU278291	37	C	0	0	0	0	2	0	0	1	0	0	0	0	0	0	0	0	0	0	0	0	0	0	0	0	3
EU278292	38	C	0	0	0	0	1	0	0	0	0	0	0	1	0	0	0	0	0	0	0	0	0	0	0	0	2
EU278293	39	C	0	0	0	0	1	0	0	0	0	0	0	0	0	0	0	0	0	0	0	0	0	0	0	0	1
EU278294	40	C	0	0	0	0	1	0	0	0	0	0	0	0	0	0	0	0	0	0	0	0	0	0	0	0	1
EU278295	41	C	0	0	0	0	1	0	0	0	0	0	0	0	0	0	0	0	0	0	0	0	0	0	0	0	1
EU278296	42	C	0	0	0	0	1	0	0	0	0	0	0	0	0	0	0	0	0	0	0	0	0	0	0	0	1
EU278297	43	C	0	0	0	0	5	0	0	0	0	1	0	0	0	0	0	0	0	0	0	0	0	0	0	0	6
EU278298	44	C	0	0	0	0	2	0	0	0	0	0	0	0	0	0	0	0	0	0	0	0	0	0	0	0	2
EU278299	45	C	0	0	0	0	1	0	0	1	0	0	0	0	0	0	0	0	0	0	0	0	0	0	0	0	2
EU278300	46	C	0	0	0	0	1	0	0	0	0	0	0	0	0	0	0	0	0	0	0	0	0	0	0	0	1
EU278301	47	C	0	0	0	0	1	0	0	0	0	0	0	0	0	0	0	0	0	0	0	0	0	0	0	0	1
EU278302	48	C	0	0	0	0	1	0	0	0	0	0	0	0	0	0	0	0	0	0	0	0	0	0	0	0	1
EU278303	49	B	0	0	0	0	1	0	0	0	0	0	0	0	0	0	0	0	0	0	0	0	0	0	0	0	1
EU278304	50	C	0	0	0	0	1	0	0	0	0	0	0	0	0	0	0	0	0	0	0	0	0	0	0	0	1
EU278305	51	C	0	0	0	0	0	16	0	0	0	0	0	0	0	0	0	0	0	0	0	0	0	0	0	0	16
EU278306	52	C	0	0	0	0	0	50	0	0	0	0	0	0	0	0	1	0	0	0	0	0	0	0	0	0	51
EU278307	53	A	0	0	0	0	0	7	0	0	0	0	0	0	0	0	0	0	0	0	0	0	0	0	0	0	7
EU278308	54	C	0	0	0	0	0	5	0	0	0	0	0	0	0	0	0	0	0	0	0	0	0	0	0	0	5
EU278309	55	A	0	0	0	0	0	3	0	0	0	0	0	0	0	0	0	0	0	0	0	0	0	0	0	0	3
EU278310	56	A	0	0	0	0	0	0	13	0	0	0	0	0	0	0	0	0	0	0	0	0	0	0	0	0	13
EU278311	57	C	0	0	0	0	0	0	1	0	0	0	0	0	0	0	0	0	0	0	0	0	0	0	0	0	1
EU278312	58	C	0	0	0	0	0	0	1	0	0	0	0	0	0	0	0	0	0	0	0	0	0	0	0	0	1
EU278313	59*	C	0	0	0	0	0	0	0	0	0	0	0	0	42	6	69	0	0	0	0	0	0	0	0	0	117
EU278314	60	A	0	0	0	0	0	0	0	0	0	0	0	0	7	7	0	0	0	34	0	15	0	0	0	0	63
EU278315	61	C	0	0	0	0	0	0	0	0	0	0	0	0	0	14	0	0	0	0	0	0	0	0	0	0	14
EU278316	62	C	0	0	0	0	0	0	0	0	0	0	0	0	0	2	1	0	0	0	0	0	0	0	0	0	3
EU278317	63	C	0	0	0	0	0	0	0	0	0	0	0	0	0	0	17	0	0	0	0	0	0	0	0	0	17
EU278318	64	C	0	0	0	0	0	0	0	0	0	0	0	0	0	0	3	0	0	0	0	0	0	0	0	0	3
EU278319	65	B	0	0	0	0	0	0	0	0	0	0	0	0	0	0	0	6	8	3	0	0	0	0	0	0	17
EU278320	66	C	0	0	0	0	0	0	0	0	0	0	0	0	0	0	3	0	0	0	0	0	0	0	0	0	3
EU278321	67	B	0	0	0	0	0	0	0	0	0	0	0	0	0	0	0	0	0	0	115	0	0	0	0	0	115
EU278322	68	A	0	0	0	0	0	0	0	0	0	0	0	0	0	0	0	0	0	0	0	25	0	0	0	0	25
EU278323	69	A	0	0	0	0	0	0	0	0	0	0	0	0	0	0	0	0	0	0	0	43	0	0	0	0	43
EU278324	70	B	0	0	0	0	0	0	0	0	0	0	0	0	0	0	0	0	0	0	0	0	19	0	1	0	20
EU278325	71	B	0	0	0	0	0	0	0	0	0	0	0	0	0	0	0	0	0	0	0	0	1	30	0	0	31
EU278326	72	B	0	0	0	0	0	0	0	0	0	0	0	0	0	0	0	0	0	0	0	0	1	0	1	0	2
GQ293462	73	B	0	0	0	0	0	0	0	0	1	0	0	0	0	0	0	0	0	0	0	0	0	0	0	0	1
GQ293463	74	B	0	0	0	0	0	0	0	0	18	0	8	0	0	0	0	0	0	0	0	0	0	0	0	0	26
GQ293464	75	B	0	0	0	0	0	0	0	0	1	0	2	0	0	0	0	0	0	0	0	0	0	0	0	0	3
GQ293465	76	B	0	0	0	0	0	0	0	0	0	0	1	0	0	0	0	0	0	0	0	0	0	0	0	0	1
GQ293466	77	B	0	0	0	0	0	0	0	0	0	0	1	0	0	0	0	0	0	0	0	0	0	0	0	0	1
GQ293467	78	B	0	0	0	0	0	0	0	0	0	0	0	0	0	0	0	4	0	0	0	0	0	0	0	0	4
GQ293468	79	B	0	0	0	0	0	0	0	0	0	0	0	0	0	0	0	1	0	0	0	0	0	0	0	0	1
GQ293469	80	B	0	0	0	0	0	0	0	0	0	0	0	0	0	0	0	0	1	0	0	0	0	0	0	0	1

GenBank Number	Haplotype	Clade	EPC	FCH	FPE	FMO	FPM	GCA	IPA	IBU	IBA	ICB	IWE	ICW	MAR'93	MAR'04	PCI	PDL	RAB	SJB	SCZ	SRL	SFM	SFN	SFX	SRP	Total
GQ293470	81	B	0	0	0	0	0	0	0	0	0	0	0	0	0	0	0	0	0	0	0	0	0	0	0	20	20
GQ293471	82	C	0	1	0	0	0	0	0	0	0	0	0	0	0	0	0	0	0	0	0	0	0	0	0	0	1
GQ293472	83	C	0	1	5	0	0	0	0	0	0	0	0	0	0	0	0	0	0	0	0	0	0	0	0	0	6
GQ293473	84	C	0	1	0	0	0	0	0	0	0	0	0	0	0	0	0	0	0	0	0	0	0	0	0	0	1
GQ293474	85	C	0	0	1	0	0	0	0	0	0	0	0	0	0	0	0	0	0	0	0	0	0	0	0	0	1
GQ293475	86	C	0	0	5	0	0	0	0	0	0	1	0	0	0	0	0	0	0	0	0	0	0	0	0	0	6
GQ293476	87	C	0	0	1	0	0	0	0	0	0	0	0	0	0	0	0	0	0	0	0	0	0	0	0	0	1
GQ293477	88	C	0	0	3	0	0	0	0	0	0	0	0	1	0	0	0	0	0	0	0	0	0	0	0	0	4
GQ293478	89	C	0	0	1	0	0	0	0	0	0	0	0	0	0	0	0	0	0	0	0	0	0	0	0	0	1
GQ293479	90	C	0	0	2	0	0	0	0	0	0	0	0	0	0	0	0	0	0	0	0	0	0	0	0	0	2
GQ293480	91	C	0	0	1	0	0	0	0	0	0	0	0	1	0	0	0	0	0	0	0	0	0	0	0	0	2
GQ293481	92	C	0	0	5	0	0	0	0	0	0	0	0	0	0	0	0	0	0	0	0	0	0	0	0	0	5
GQ293482	93	C	0	0	1	0	0	0	0	2	0	0	0	0	0	0	0	0	0	0	0	0	0	0	0	0	3
GQ293483	94	C	0	0	1	0	0	0	0	0	0	0	0	0	0	0	0	0	0	0	0	0	0	0	0	0	1
GQ293484	95	C	0	0	2	0	0	0	0	0	0	0	0	0	0	0	0	0	0	0	0	0	0	0	0	0	2
GQ293485	96	C	0	0	1	0	0	0	0	0	0	0	0	0	0	0	0	0	0	0	0	0	0	0	0	0	1
GQ293486	97	C	0	0	1	0	0	0	0	0	0	0	0	0	0	0	0	0	0	0	0	0	0	0	0	0	1
GQ293487	98	C	0	0	0	0	0	0	0	4	0	0	0	0	0	0	0	0	0	0	0	0	0	0	0	0	4
GQ293488	99	C	0	0	0	0	0	0	0	1	0	0	0	0	0	0	0	0	0	0	0	0	0	0	0	0	1
GQ293489	100	C	0	0	0	0	0	0	0	1	0	0	0	0	0	0	0	0	0	0	0	0	0	0	0	0	1
GQ293490	101	C	0	0	0	0	0	0	0	0	1	0	0	0	0	0	0	0	0	0	0	0	0	0	0	0	1
GQ293491	102	C	0	0	0	0	0	0	0	0	0	1	0	0	0	0	0	0	0	0	0	0	0	0	0	0	1
GQ293492	103	C	0	0	0	0	0	0	0	0	0	0	0	3	0	0	0	0	0	0	0	0	0	0	0	0	3
GQ293493	104	C	0	0	0	0	0	0	0	0	0	0	0	4	0	0	0	0	0	0	0	0	0	0	0	0	4
GQ293494	105	C	0	0	0	0	0	0	0	0	0	0	0	1	0	0	0	0	0	0	0	0	0	0	0	0	1
GQ293495	106	C	0	0	0	0	0	0	0	0	0	0	0	0	0	0	0	0	2	0	0	0	0	0	0	0	2
Total			98	20	86	60	73	81	54	15	21	7	13	30	49	29	94	12	11	72	116	83	82	52	25	20	1203

*Haplotypes 59b (GQ293496) and 59c (GQ293497) are identical to Haplotype 59, except that they differ in sequence length due to indels. Haplotypes 59b and 59c were not considered in analyses
