# Supplementary material for: Progressive colonization and restricted gene flow shape island-dependent population structure in Galápagos marine iguanas (Amblyrhynchus cristatus)
Source: BMC Evol Biol. 2009 Dec 22;9:297. doi: 10.1186/1471-2148-9-297 (PMC2807874; doi:10.1186/1471-2148-9-297)
Supplement: Additional file 4 — Table S3: General characteristics of the three mitochondrial clades of Galápagos marine iguanas. [file 1471-2148-9-297-S4.DOC]

**Table S3.** General characteristics of the three Galápagos marine iguana clades. (a) Genetic distances among haplotypes within clades under the Kimura 2-parameter (K2P) model. Maximum distances are also referred to in number of substitutions considering transitions (Ti) only, transversions only (Tv), and both transitions and transversions (Total). (b) Average (above diagonal) and maximum (below diagonal) K2P distances between the three marine iguana clades

(a)

| **Clade** | **No. of individuals** | **No. of haplotypes** | **No. of singletons** | **Within-clade pairwise distances** | | |
| --- | --- | --- | --- | --- | --- | --- |
|  |  |  |  | **Average** | **Max.** | **Max. Ti/Tv/Total** |
| A | 155 | 7 | 1 | 0.0045 | 0.0077 | 7/2/9 |
| B | 541 | 30 | 8 | 0.0047 | 0.0103 | 12/4/12 |
| C | 507 | 69 | 26 | 0.0040 | 0.0077 | 8/2/9 |
| Total | 1203 | 106 | 35 | 0.0072 | 0.0164 | 18/4/19 |

**(b)**

|  | **Avg. between-clade pairwise distances** | | |
| --- | --- | --- | --- |
| **Clade** | **A** | **B** | **C** |
| **A** | - | 0.0127 | 0.0105 |
| **B** | 0.0164 | - | 0.0099 |
| **C** | 0.0155 | 0.0146 | - |
