# Supplementary material for: Progressive colonization and restricted gene flow shape island-dependent population structure in Galápagos marine iguanas (Amblyrhynchus cristatus)
Source: BMC Evol Biol. 2009 Dec 22;9:297. doi: 10.1186/1471-2148-9-297 (PMC2807874; doi:10.1186/1471-2148-9-297)

Suppl. Figure 1: Estimation of the true number of genetic clusters detected by the program *Structure* by calculating ΔK=m[|L″K|]/s[L(K)] following the approach of Evanno et al. [44] for values of K from 1-25. The local maximum is found for K=20.

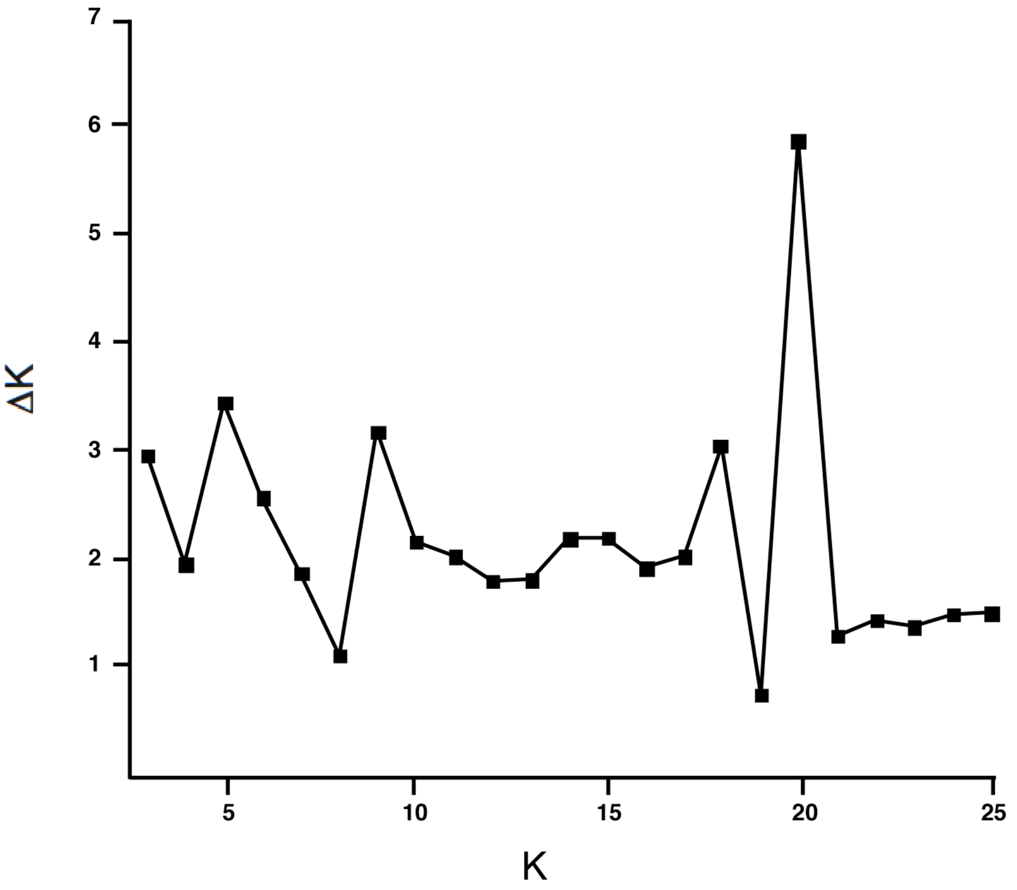

Supplement: Additional file 5 — Figure S1: Estimation of genetic clusters according to Evanno et al. [file 1471-2148-9-297-S5.PDF]
