# Supplementary material for: Progressive colonization and restricted gene flow shape island-dependent population structure in Galápagos marine iguanas (Amblyrhynchus cristatus)
Source: BMC Evol Biol. 2009 Dec 22;9:297. doi: 10.1186/1471-2148-9-297 (PMC2807874; doi:10.1186/1471-2148-9-297)
Supplement: Additional file 6 — Table S4: Best found partitions according to BAPS v3.2. [file 1471-2148-9-297-S6.DOC]

**Table S4:** List of sizes of ten best visited partitions and corresponding log (ml) values according to the program BAPS v3.2 . The best partition found corresponds to 20 genetic clusters.

| Number of genetic clusters | Log (ml) values |
| --- | --- |
| 20 | -6.091e^+^004 |
| 20 | -6.091e^+^004 |
| 20 | -6.091e^+^004 |
| 20 | -6.091e^+^004 |
| 19 | -6.092e^+^004 |
| 19 | -6.092e^+^004 |
| 19 | -6.093e^+^004 |
| 19 | -6.093e^+^004 |
| 18 | -6.093e^+^004 |
| 18 | -6.093e^+^004 |
