# Supplementary material for: Progressive colonization and restricted gene flow shape island-dependent population structure in Galápagos marine iguanas (Amblyrhynchus cristatus)
Source: BMC Evol Biol. 2009 Dec 22;9:297. doi: 10.1186/1471-2148-9-297 (PMC2807874; doi:10.1186/1471-2148-9-297)
Supplement: Additional file 7 — Figure S2: Scatter plot FST(based on microsatellite loci) versus possible dispersal distance. [file 1471-2148-9-297-S7.PDF]

Supplementary Figure 2: Scatter plot and regression analysis of possible dispersal distance (see Method section for details) and genetic distance (F_{ST} based on microsatellite loci).

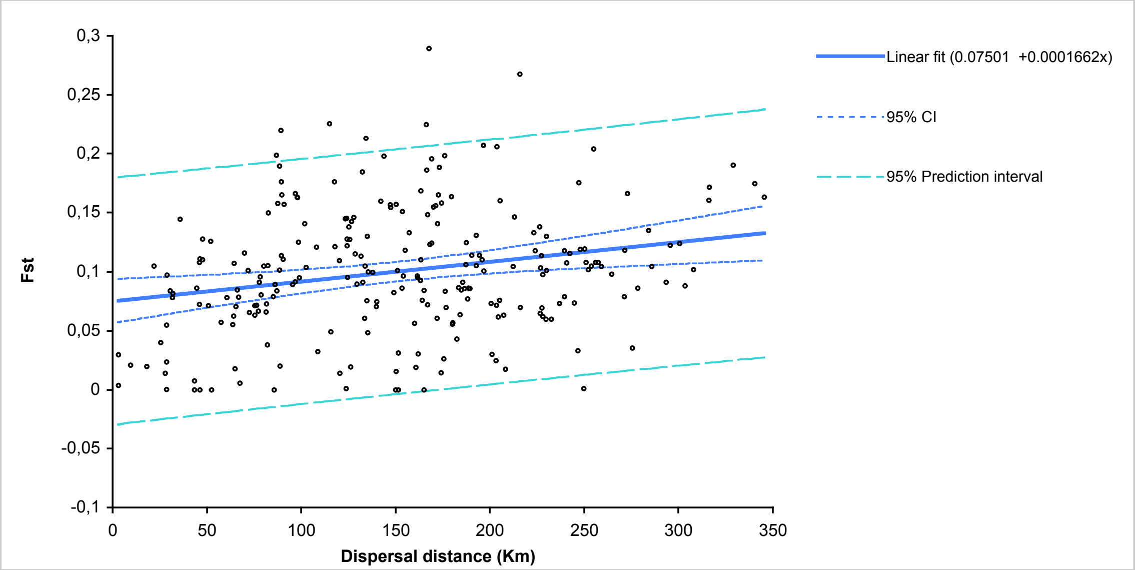

A: Between island populations of marine iguanas ($R^2 = 0.05$; $P < 0.0001$).

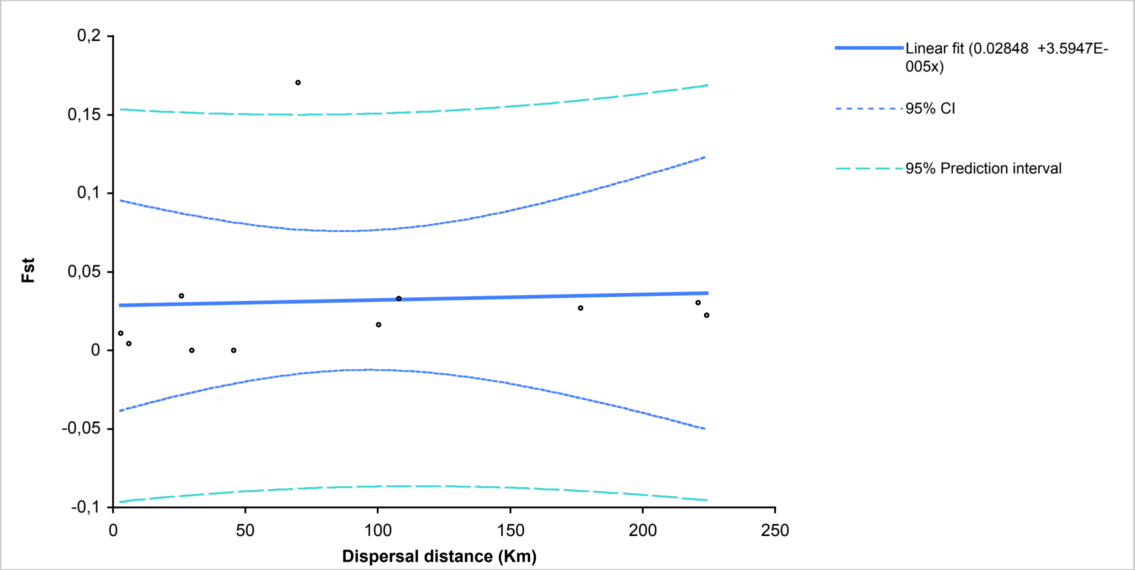

B: Between within-island populations ($R^2 = 0.00$; $P = 0.25$).
