# Supplementary material for: Progressive colonization and restricted gene flow shape island-dependent population structure in Galápagos marine iguanas (Amblyrhynchus cristatus)
Source: BMC Evol Biol. 2009 Dec 22;9:297. doi: 10.1186/1471-2148-9-297 (PMC2807874; doi:10.1186/1471-2148-9-297)

Suppl. Figure 3: Mismatch distributions of Galápagos marine iguana mtDNA CR haplotypes. (a) clade C individuals from western islands; (b) clade B individuals from eastern and central islands; (c) clade C individuals from northern islands; (d) the entire marine iguana dataset. The line with closed circles is the observed distribution of pairwise differences and the smooth line is the expected distribution under the sudden expansion model [90]. The sum of squared deviation (*SSD*) between the observed and expected distributions, the raggedness statistic (*r*) [92], and their corresponding *p*-values are reported. The values of the expansion model parameters τ (mutational time), θ_0 and θ_1 are also shown as well as the 95% confidence interval of the estimate of τ.

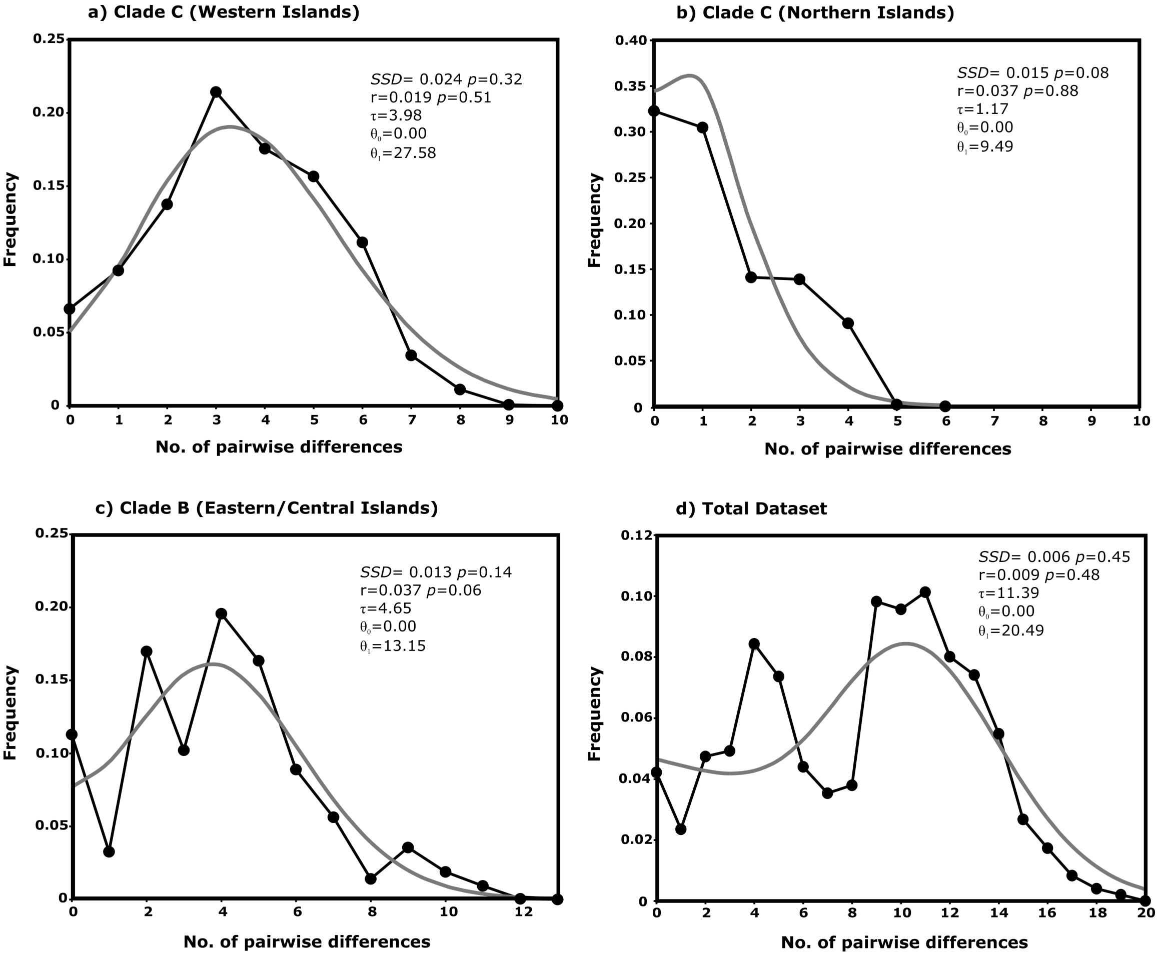

Supplement: Additional file 8 — Figure S3: Mismatch distribution of Galápagos marine iguana D-loop haplotypes. [file 1471-2148-9-297-S8.PDF]
