# Supplementary material for: Progressive colonization and restricted gene flow shape island-dependent population structure in Galápagos marine iguanas (Amblyrhynchus cristatus)
Source: BMC Evol Biol. 2009 Dec 22;9:297. doi: 10.1186/1471-2148-9-297 (PMC2807874; doi:10.1186/1471-2148-9-297)
Supplement: Additional file 9 — Table S5: Intra locus specific k-values of thirteen microsatellite loci of Galápagos marine iguana populations. [file 1471-2148-9-297-S9.DOC]

**Table S5:** Intra-locus specific k-values of thirteen microsatellite loci for Galápagos marine iguana populations. Populations were sampled in 1991/1993*^a^* and/or 2004*^b^*. Corresponding p-values are provided by Table 1.

| Population | EPC (Punta Cevallos)^a,b^ | FCH (Capo Hammond)^a^ | FPE (Punta Espinosa)^a,b^ | FPM (Punta Mangle)^a,b^ | FMO (Punta Montura)^a^,b | GCA (Campamente)^a,b^ | IBU (Bahia Urvina)^b^ | ICW (Caleta Webb)^a,b^ |
| --- | --- | --- | --- | --- | --- | --- | --- | --- |
| MIGE2 | 7,020290128 | -2,866211475 | -182,5180288 | -211,6557622 | -6,589185595 | -2,353332635 | -9,248051866 | -153,7164461 |
| MIG-E3 | -18,17745846 | -51,65770019 | -56,89857194 | -40,27970636 | -7,235087362 | 4,078457504 | 8,637891756 | -30,3618421 |
| MIG-E4 | -116,4357416 | -381,3664649 | -634,4371187 | -44,28431819 | 371,3801628 | -1023,979301 | -1646,621133 | -336,6745673 |
| MIG-E6 | 181,1214516 | -10,04234147 | 1,773255377 | 27,03543335 | 101,8571193 | 2,985312733 | 18,08628256 | 6,612416636 |
| MIG-E8 | -7,414705841 | 1,675598321 | 1,302389999 | -11,32726576 | -46,836805 | 10,78139143 | 3,229751093 | -4,042115587 |
| MIG-E10 | -41,60749782 | -42,77473847 | -33,08193446 | -24,63386949 | 2,802792662 | -8,476221961 | -12,54227646 | -53,22230318 |
| MIG-E11 | 0,72183977 | -15,09674864 | -8,312352779 | -27,36608947 | -6,589690351 | 1,79189306 | -103,3095777 | -4,870867651 |
| MIG-E12 | 600,6623523 | 1012,695848 | 753,036962 | 575,8763194 | 363,9208921 | 5,934966563 | 739,2378967 | 915,2155481 |
| MIG-E13 | 26,5938748 | -1,003772421 | 19,75488225 | -97,38265216 | 36,11603087 | -7,735606818 | -6,562931545 | -221,7383703 |
| MIG-E14 | -186,5081732 | -2095,226622 | -1363,913525 | -805,2803454 | 497,771138 | 1,061544216 | -46,58690831 | 89,39714107 |
| MIG-E15 | 3,652867153 | 0 | -56,03944559 | 97,44596383 | -11,74270983 | -1,151285526 | 201,8368222 | 193,4382512 |
| MIG-E16 | -6,869738952 | 2,619327039 | 0,977290218 | -3,175994913 | -2,113290398 | -0,777797963 | -1,944070946 | -4,141646462 |
| Am(GT)4 | -3,650256831 | -18,34911585 | -4,391174028 | -1,697397281 | 1,548947738 | 0,231863384 | -14,97705785 | 17,43812591 |
|  |  |  |  |  |  |  |  |  |
|  |  |  |  |  |  |  |  |  |
|  |  |  |  |  |  |  |  |  |
| Population | IBA (Cerro Ballena)^b^ | IPA (Punta Albemarle)^a,b^ | MBN (1993) | MBN (2004) | PCI (Caleta Ibetson)^a,b^ | PDL (Dumb Landing)^a^ | RAB (No name)^b^ | SRL (Loberia)^a,b^ |
| MIGE2 | -241,1870927 | -242,1069723 | -61,30959165 | -35,74979928 | -6,235345428 | -0,3375 | -14,49149381 | 0,245272995 |
| MIG-E3 | -22,54617656 | -51,70339643 | 5,344996222 | 1,723881512 | 19,71877882 | -41,94513072 | 0,905734262 | 0,631114582 |
| MIG-E4 | -322,0486961 | -512,9815138 | -231,6923153 | -509,4134913 | -490,5184564 | -1346,409837 | -183,6445857 | -288,5223676 |
| MIG-E6 | 0,116897058 | 19,40857401 | -18,8057925 | -33,02236176 | 10,62288787 | 78,06369281 | -0,584259546 | 0,307756864 |
| MIG-E8 | -1,666458792 | 3,388671384 | 14,20388644 | 18,63262805 | -9,434008353 | 0,637124183 | -0,501274338 | -2,458179164 |
| MIG-E10 | -51,16312421 | -29,38713247 | 5,525202011 | 1,070616273 | 0,718611836 | 24,36075163 | -42,91335759 | 2,982540187 |
| MIG-E11 | 11,39362607 | 0,660587658 | 67,81259596 | 260,0534809 | 169,2924194 | -4,955081699 | 2,855772102 | -12,25700683 |
| MIG-E12 | 460,3856532 | 384,1665291 | -201,1581415 | -243,5713768 | -371,0155406 | -4,298529412 | 448,4443308 | 12,34338841 |
| MIG-E13 | 2,46515394 | -317,7970311 | 0,013612912 | 8,184587084 | 6,013628413 | -2,73996732 | 9,238485552 | 35,53966808 |
| MIG-E14 | -1480,623745 | 796,2680823 | 1367,468012 | 1064,062304 | -633,8703304 | 2003,982908 | 127,976383 | 12,28935039 |
| MIG-E15 | 716,4384346 | -54,47842272 | 34,98541572 | -20,66522363 | 358,3325964 | 32,97506536 | 507,9324649 | 5,54451377 |
| MIG-E16 | -14,4807865 | 1,003825278 | -8,531028433 | -4,691349886 | -4,222875808 | 2,111764706 | -0,092777778 | -7,588773698 |
| Am(GT)4 | -0,290017366 | -7,186580348 | 0,085244949 | -0,634310371 | 14,91112309 | -1,57996732 | -0,140751634 | 15,00533438 |
|  |  |  |  |  |  |  |  |  |
| Population | SRP (Punta Pitt)^a^ | SCZ (Estacion/Camaño)^a,b^ | SFN (North)a | SFM (Miedo)^a,b^ | SFX (Bahia Paraiso)^b^ | SJB (James Bay)^a,b^ |  |  |
| MIGE2 | 0,398856036 | -1,164473841 | -11,34751666 | -12,25123487 | -16,44037086 | -116,3119486 |  |  |
| MIG-E3 | 18,79233914 | -8,474476123 | -3,199534733 | -2,242339192 | -2,537390967 | -0,916954313 |  |  |
| MIG-E4 | -780,2190849 | -952,764595 | -182,8145141 | -133,4241838 | 192,2473824 | -1425,396765 |  |  |
| MIG-E6 | 1,712538653 | 14,84542818 | 33,21991426 | 29,49248613 | 21,70357456 | -21,60116265 |  |  |
| MIG-E8 | 0,015387542 | -11,52908822 | -2,741089432 | -20,1084464 | -41,79297079 | -8,014420366 |  |  |
| MIG-E10 | 6,73405213 | -21,62894661 | -26,17478776 | -34,67956541 | 1,570194457 | 83,5535828 |  |  |
| MIG-E11 | 1,09885167 | -4,253287352 | 0,704039582 | -2,828255245 | 1,112096955 | -95,88159807 |  |  |
| MIG-E12 | -0,946057182 | -386,6446914 | -288,2865084 | -302,8651314 | -364,7131815 | 335,2069368 |  |  |
| MIG-E13 | 77,37960635 | 52,47209633 | -4,877673097 | 28,12974218 | -30,49403979 | -2,865922798 |  |  |
| MIG-E14 | 1047,676076 | 771,0750147 | 31,75648127 | -82,86179554 | -92,11907859 | 468,4230366 |  |  |
| MIG-E15 | -25,35612571 | 203,5515982 | -28,34398272 | -18,62811592 | -27,0510788 | -118,558785 |  |  |
| MIG-E16 | -3,068049376 | 0,382284556 | 0,13364557 | 0,102765778 | 0,129886615 | -0,491179841 |  |  |
| Am(GT)4 | -7,800135135 | 15,22169823 | 1,629680645 | 40,12143493 | 32,69219004 | -9,691980688 |  |  |
