# Supplementary material for: Progressive colonization and restricted gene flow shape island-dependent population structure in Galápagos marine iguanas (Amblyrhynchus cristatus)
Source: BMC Evol Biol. 2009 Dec 22;9:297. doi: 10.1186/1471-2148-9-297 (PMC2807874; doi:10.1186/1471-2148-9-297)
Supplement: Additional file 10 — Figure S4: Scatter plot with regression analysis of island perimeter as measured by NOAA GIS resource (World Vector Coastline) vs genetic diversity values. [file 1471-2148-9-297-S10.DOC]

**Suppl. Figure 4:** Scatter plot with regression analysis of island perimeter as measured by

NOAA GIS resource (World Vector Coastline) *vs* genetic diversity values.


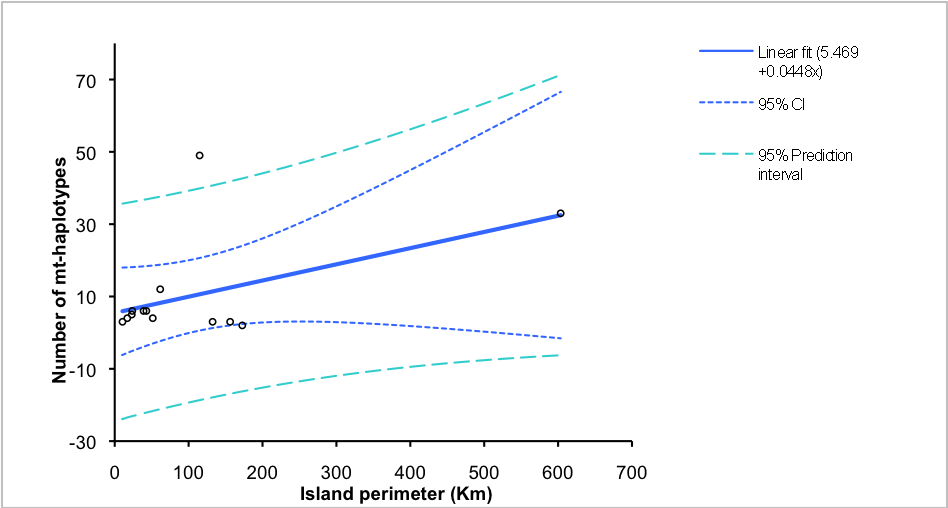


A: Island perimeter *vs* number of D-loop haplotypes per island (R2 = 0.25; P = 0.08).


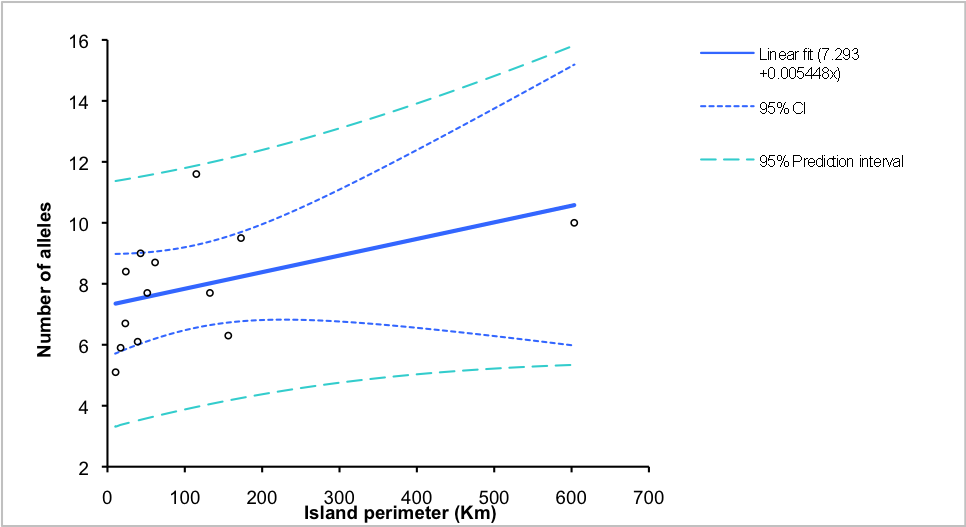


B: Island perimeter *vs* number of average microsatellite loci alleles (R2 = 0.21; P = 0.11).
